# Occupational Exposure to Resorcinol and Thyroid-Disrupting Effects: Protocol for an Exploratory Field Study in French Hairdressers

**DOI:** 10.2196/65833

**Published:** 2026-03-31

**Authors:** Anca Radauceanu, Amandine Cambrai-Erb, Benedicte Adet, Marie-Isabelle Nioule, Flavien Denis, Romain Pons

**Affiliations:** 1 Department of Occupational Epidemiology, French Research and Safety Institute for the Prevention of Occupational Accidents and Diseases (INRS), 1 rue du Morvan, Vandoeuvre-lès-Nancy Cedex, 54519, France, 33 0687248344; 2 Department of Toxicology and Biomonitoring, French Research and Safety Institute for the Prevention of Occupational Accidents and Diseases (INRS), Vandoeuvre-lès-Nancy Cedex, France

**Keywords:** resorcinol, hairdresser, endocrine-disrupting chemicals, occupational exposure, thyroid gland, biomonitoring, cosmetics, hair products, thyroid disruptors, disruptors of thyroid hormones

## Abstract

**Background:**

All around the world, the hairdressing sector constitutes a major occupational group, including about 90% women, most of whom are of reproductive age. Hairdressers are continuously exposed to numerous chemicals used in hair products, including endocrine-disrupting compounds such as resorcinol, parabens, phthalates, and UV filters. Few biomonitoring studies have explored occupational exposure to endocrine disruptors in hairdressers, and no data were found on their impact on the thyroid hormone system. Resorcinol is an oxidative hair dye with thyroid-disrupting properties that decrease thyroid hormone synthesis and could alter neurodevelopmental functions during fetal and perinatal stages in case of maternal exposure.

**Objective:**

This study aims to assess the occupational exposure to resorcinol in French hairdressers and analyze the relationship with biological thyroid parameters, taking into account the occupational exposure to other potential thyroid disruptors (parabens and UV filters like benzophenone and cinnamates).

**Methods:**

An exposed-unexposed cross-sectional study is proposed involving female hairdressers aged 18 to 45 years (working in hair salons) compared to occupationally unexposed controls (employed in office activities), who are recruited within 14 French occupational health centers. The hairdressers are followed during a 5-day working week to assess exposure data at both the individual level and the salon level. Urinary samples for the measurement of thyroid disruptors (resorcinol, parabens, metabolites of ethylhexyl methoxycinnamate, and benzophenone-3) are collected at 6 time points (before the day 1 shift, before and after the day 3 and day 4 shifts, and before the day 5 shift). Daily work tasks and use of hair products are self-reported within the workplace, and a complete inventory of hair products within the salon is carried out. Thyroid disruption effects are assessed by measuring blood thyroid parameters: triiodothyronine, thyroxine, thyroid-stimulating hormone, thyroglobulin, thyroperoxidase, and thyroglobulin antibodies. To assess nonoccupational exposure to thyroid disruptors and other confounding factors, information on sociodemographic data, place of residence, food and tobacco consumption, personal use of care products, professional career, and medical history is collected through questionnaires. Regarding statistical analysis, urinary samples from hairdressers and controls will be compared, and adjusted multivariable models will be used to analyze health outcomes.

**Results:**

The study duration extends from 2022 to 2027. As of December 2025, 9 occupational health centers have enrolled 66 hairdressers (employed in 54 hair salons) and 30 occupationally unexposed participants.

**Conclusions:**

The results will represent the first data on occupational exposure to resorcinol in France and its relationship with thyroid hormones in hairdressers. Following a multidisciplinary approach that includes biomonitoring, epidemiology, and exposure data collection at both the hairdressers and salon levels, this study enables an in-depth assessment of exposure to the thyroid disruptors in the workplace. Together with the inventory of hair products, these results may enhance the tools for chemical risk assessment and prevention in hair salons.

## Introduction

### Principles of Cosmetic Product Regulation in the European Union: Highlight on Endocrine-Disrupting Chemicals

In the European Union, the legal framework for cosmetic products relies on the Cosmetic Products Regulation Number 1223/2009 [1]. The main purpose of the cosmetic product legislation is to ensure the safety of cosmetics for consumers through rigorous science-based regulation. More specifically, ingredient regulation is based on a “risk-based” approach, implemented through the establishment of positive and negative lists of ingredients [2]. The Scientific Committee on Consumer Safety (SCCS) provides guidance on the safety of cosmetic ingredients for consumers only, but not for professional users. In the European Union, there is no specific management of endocrine-disrupting chemicals (EDCs) in cosmetics. These substances, defined by the World Health Organization as “exogenous substances or mixtures that alter the function(s) of the endocrine system and consequently cause adverse effects in an intact organism, or its progeny, or (sub)populations” [3], are evaluated and regulated on a compound-by-compound basis by the SCCS, which provides safety assessments of cosmetic products at certain concentrations [4]. A number of restrictions can be applied when the substances are also classified as carcinogenic, mutagenic, or toxic for reproduction or considered substances of very high concern (SVHC), defined as substances that may have serious and often irreversible effects on human health and the environment. The European Union recently introduced new hazard classes to reinforce the classification, labeling, and packaging (CLP) of substances and mixtures regulation, which serves as the central piece of the chemical legislation addressing endocrine disruptors for human health and the environment [5]. Thus, 2 hazard classes and corresponding statements for endocrine disruptors have been created based on their scientific strength of evidence: known or presumed endocrine disruptors (category 1) and suspected endocrine disruptors (category 2) for both human health and the environment.

Within the European Union, 6 national competent authorities compile information about the current status of substances identified as EDCs or those under evaluation for endocrine-disrupting properties through 3 lists, which are updated at least biannually and hosted on a dedicated website [6]. The 3 endocrine disruptor lists consist of the following: list 1, substances identified as endocrine disruptors that have undergone the full evaluation of endocrine-disrupting properties; list 2, substances that are currently under evaluation; and list 3, substances considered endocrine disruptors at the national level in one of the participating Member States.

### EDCs in Hair Products: Focus on Thyroid Hormone System–Disrupting Chemicals

#### Overview

Cosmetic hair products belong to a wide spectrum of items ranging from shampoo, conditioner, and hair dyes to fragrances, solutions, or sprays for hair bleaching, perming, straightening, styling, and modeling. The endocrine disruptors in hair products are compounds that serve various functions (preservatives, hair dyeing, perfuming, UV protection, etc), and the most commonly reported endocrine disruptors in the literature are parabens, phenols, bisphenols, phthalates, cinnamates, and benzyl salicylate [7-9].

#### Brief Inventory of EDCs in Professional Hair Products

In 2020, we conducted a brief inventory of EDCs identified among the compounds of 1157 hair cosmetic products available for sale to hairdressers on professional websites [10].

In this inventory, the content of hair products revealed the presence of resorcinol in 40% of hair dyes, parabens in around 20% of styling waxes as well as gels and lacquers for men, salicylic acid in 20% of shampoos, cyclopentasiloxane in 15% of hair care products and conditioners, and butylated hydroxytoluene in 10% of hair gels or sprays. The most frequently used UV filters and absorbers were benzyl salicylate (25% of hair products), benzophenones (15%‐20% of shampoos for colored hair and styling products), and ethylhexyl methoxycinnamate (10%‐12% of hair gels, lacquers, sprays, hair care, and conditioners).

#### Thyroid Hormone System and Thyroid Dysfunction

Thyroid hormones are involved in numerous physiological processes as regulators of metabolism, bone remodeling, cardiac function, fetal neurodevelopment, and mental status [11,12]. Thyroid function is regulated through the hypothalamus-pituitary-thyroid axis in which the hypothalamus releases thyrotropin-releasing hormone to stimulate the anterior pituitary gland to produce thyroid-stimulating hormone (TSH), which in turn stimulates the production and secretion of thyroid hormones (thyroxine and triiodothyronine) from the thyroglobulin stored in the thyroid follicles.

The incidence of thyroid dysfunction has recently increased worldwide, particularly among women, since the ratio of thyroid dysfunction among women of reproductive age compared to men is 4:1 [13]. Hypothyroidism is the most frequent, followed by hyperthyroidism, which is also associated with a 4.5-fold increased risk of thyroid cancer [14]. Overall, 5% to 9% of adults have subclinical thyroid disease, and 0.8% to 7.5% of adults have overt clinical thyroid disease [13,15]. A 2014 meta-analysis of older European studies reported a mean prevalence of total thyroid dysfunction of 3.82% (0.75% for hyperthyroidism and 3.05% for hypothyroidism), while the prevalence of undiagnosed thyroid dysfunction was almost 7% [16].

In France, 3 studies estimated the prevalence of hyperthyroidism and hypothyroidism. The first study was conducted from 1994 to 1995, based on the measurement of serum thyrotropin (TSH), and estimated a prevalence of 5.7% for hyperthyroidism in participants aged between 35 and 60 years [17]. A recent study, conducted in 2020, estimated prevalences of treated hyperthyroidism and hypothyroidism, according to national health care data, at 0.17 and 4.45 per 100 inhabitants, respectively [18]. Prevalence was higher for women than for men and increased with age. Interestingly, French data on treated acquired hypothyroidism indicate a decreased incidence for both female and male participants from 2014 to 2019, for all age groups over 15 years of age [19].

Due to the serious health consequences and financial burden associated with thyroid diseases, the identification of risk factors, especially those that are modifiable, is of utmost public health interest. There is compelling evidence from in vivo, in vitro*,* and epidemiological studies that a growing number of chemicals can disrupt thyroid function and cause adverse health effects [20,21]. Thyroid-disrupting health effects from exposure to care and other consumer products have been reported, and their continuous consumption nowadays negatively affects susceptible populations, such as pregnant women and their offspring, with long-term adverse consequences, such as attention or deficit or hyperactivity disorders, autism spectrum disorders, and developmental delays in the offspring [22].

#### Thyroid-Disrupting Chemicals in Hair Products: Focus on Resorcinol

Among the compounds in hair products, parabens have been shown to have a disruptive effect on the thyroid in both experimental and human studies [21,23], whereas for benzophenone UV filters, the relevance of human exposure needs to be studied further [24]. However, increased urinary benzophenone-3 has been associated with decreased total triiodothyronine in pregnant women [25]. Regarding cinnamate UV filters, only experimental studies have indicated interferences with thyroid function [9]. Resorcinol is an oxidative hair dye (maximum allowed concentration up to 1.25%) and also an ingredient in hair lotions and shampoos (maximum allowed concentration up to 0.5%) [26]. Additionally, resorcinol is used in other industrial fields for its properties (antioxidant, antiseptic, enhancing strength of rubber and wood products, UV protection, flame retardant, colorant, and food additive). It is also found in the environment as a result of anthropogenic activities, as a metabolite of tannic acid frequently found in foods and beverages and as a component of tobacco smoke. The global resorcinol market stood at nearly 70,000 tons in 2022 and is expected to grow at a compound annual growth rate of 3.94% over the forecast period until 2032 [27].

In the professional use of resorcinol or resorcinol-containing products, the main route of exposure is skin contact, as resorcinol is a molecule of low volatility. Nevertheless, inhalation may occur if resorcinol is aerosolized, as well as oral exposure through hand-to-mouth contact.

After absorption, resorcinol is mainly excreted in urine, and urinary resorcinol serves as a valuable marker of occupational exposure [28]. Since resorcinol has a short half-life and no tissue bioaccumulation, it is expected that nearly all absorbed resorcinol will be excreted in urine within 24 hours after exposure.

**Figure 1.**
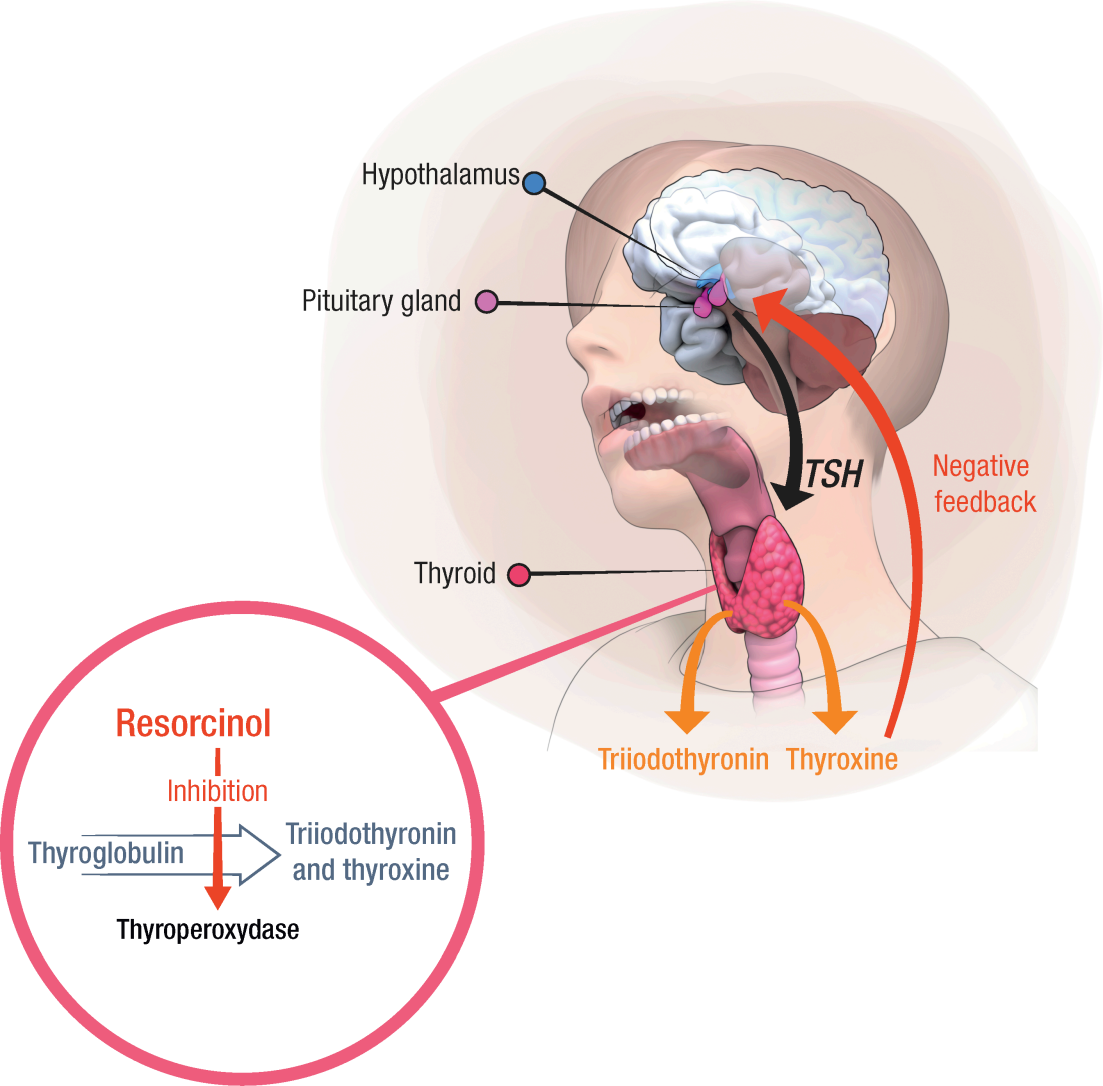
Thyroid-disrupting mechanism of resorcinol involving thyroperoxidase inhibition leading to impaired iodination of thyroglobulin in the thyroid gland, reduced thyroid hormones (triiodothyronine and thyroxine) synthesis, and compensatory increase in thyroid-stimulating hormone (TSH) levels.

In 2020, the French Agency for Food, Environmental and Occupational Health & Safety submitted a proposal for identifying resorcinol as an SVHC based on the criteria set out in the European regulation REACH (Registration, Evaluation, Authorization, and Restriction of Chemicals). This report was based on the scientific evidence of probable serious effects on human health in relation to its thyroid-disrupting potential, as shown in numerous in vitro and in vivo studies [29]. Resorcinol acts principally via the inhibition, in a dose-dependent manner, of the key enzyme involved in thyroid hormone synthesis, thyroperoxidase, resulting in the blockage of thyroid hormone synthesis and decreased serum thyroxine level. Consistent with thyroperoxidase inhibition, resorcinol inhibits intrathyroid iodine uptake and organification in humans, rodents, and more recently, fishes and lizards, leading to clinical hypothyroidism [29,30]. Low thyroid hormone levels trigger a negative feedback mechanism mediated by the hypothalamic thyrotropin-releasing hormone, which stimulates the synthesis and release of TSH in the pituitary gland to compensate for insufficient levels of thyroid hormones (Figure 1). Compensatory mechanisms within the thyroid gland, in response to the increase in TSH (thyroid hyperplasia, thyroglobulin depletion, increased iodine absorption), are unable to restore normal thyroid function because of the direct inhibition of thyroperoxidase.

Since resorcinol has been used as a dermally applied ointment to treat mainly skin ulcers and, more rarely, acne, clinical hypothyroidism has been reported in several human cases after repeated application, as well as the reversal of symptoms and goiter after cessation of the exposure [29]. In animals, effects on the thyroid were consistent with human effects, together with histopathological changes in the thyroid and probable reproductive and neurodevelopmental toxicity. For humans, the relationship between the inhibition of thyroperoxidase, the decrease in maternal circulating thyroid hormones, and prenatal and postnatal neurodevelopmental alterations in offspring is considered to be established with a high level of evidence. Finally, even though resorcinol is a chemical with clear thyroid-disrupting properties in humans, its thyroid-disrupting effects have not been explored in the context of occupational exposure.

### Hairdressing, Occupational Exposure to Endocrine-Disrupting Chemicals, and Health End Points

Around the world, the hairdressing sector constitutes a major occupational group employing a majority of women, many of whom are of reproductive age [31]. In France, in 2022, there were 180,000 hairdressers, and about 90% of them were women [32]. This occupational group is continuously exposed to numerous chemical ingredients found in hairdressing products, as well as to cleaning products used in the workplace [33]. In contrast to most customers who use cosmetics only for a few minutes each day, hairdressers may be exposed to these products for up to 8 hours per day and 5 or 6 days per week, throughout their careers. The average frequency of use by consumers, therefore, severely underestimates the exposure of hairdressers to daily skin contact with hairdressing products [34], and the safety assessment for consumer use differs from that for professional use. For instance, in an SCCS report in the Netherlands undertaken on 56 hair dyes, more than 37% of the products were considered safe for consumers but exhibited margins of safety that were considered as risky for professionals [35]. The routes of exposure differ depending on the type and use of the products. For example, during the hair coloring stages, powder may be inhaled during mixing, skin contact may occur when the mixtures are applied, and gases or vapor could be inhaled due to evaporation [36]. Workplace characteristics (ventilation and size of the salon), working techniques (haircutting after applying product mixtures and use of protective equipment), hairdressers’ information, and prevention education are also relevant factors influencing the exposure of hairdressers [36].

The occupational hazards of cutaneous and respiratory allergic diseases in hairdressing are well described, but differences have been reported between information declared in surveys and work observations, particularly regarding the wearing of gloves, suggesting their use was not systematic during the steps of hair dyeing following application, such as washing or rinsing and haircutting [36]. Resorcinol was therefore found in most handwash samples from hairdressers after the application of hair dye, as well as after the haircut that follows hair dye, once gloves had been removed [37,38].

Overall, the inappropriate use of gloves during tasks (sharing and reusing, wearing jewelry, unsuitable cheap gloves), inadequate skin protection, working in wet environments, likely present to a large extent in hairdressers, impaired epidermal barrier function, aggregated exposure (exposure to the same ingredient via different products) among others, and facilitated the penetration of hazardous substances into the skin, consequently increasing the risk of local and possibly systemic adverse effects [34]. Recent studies assessing chemical exposure among hairdressers are limited and have mainly focused on air contaminants in hairdressing salons, such as volatile organic compounds, phthalates, or particulate matter [31,39]. Furthermore, there are few urinary biomonitoring studies that enable an evaluation of overall exposure, including the cutaneous route of exposure to hair products [31,34]. Among these studies, few have explored occupational exposure to endocrine disruptors in hairdressers. In 2019, Quiros-Alcala et al [31] reviewed 2 studies, one focusing on urinary resorcinol among hairdressers and the other on urinary phthalate among hairdressing apprentices. In the first study, which aimed to quantify environmental and occupational exposure to resorcinol in urine in Finland, no difference was observed between hairdressers and nonoccupationally exposed women [40]. Nevertheless, concentration levels of resorcinol were higher in postshift samples compared to the morning sample taken after at least 1 day off from work. In the second study, urinary phthalate biomarker levels were higher in Slovakian hairdressing apprentices compared to controls, and the latter were associated with negative outcomes in pulmonary function [41]. More recently, Kolena et al [42] published a second Slovakian study that confirmed significant exposure to phthalates related to hairdressing among apprentice hairdressers and found a relationship between lung function tests and anthropometric parameters adjusted for occupational exposure to phthalates. The authors suggested that exposure to phthalates could affect the body composition related to fat and fat-free mass index, which could secondarily affect lung function. In comparison to controls, US hairdressers serving an ethnically diverse clientele had concentrations of urinary phthalate metabolites 10 times higher after their shift [43]. These hairdressers were also exposed to 24 additional chemicals with median peak areas twice as high, including methylparaben, ethylparaben, propylparaben, and 2-naphthol [44].

As the hairdressing workforce includes a large majority of women of childbearing age, most of the research has focused on reproductive effects. Chemicals in hair products have changed significantly over time, but an updated review including epidemiological studies published in the last 2 decades still reported scarce adverse reproductive outcomes (poor neonatal health indicators and perinatal adverse events) [45]. A recent Brazilian study highlighted a higher number of premature deaths due to reproductive system cancers in female hairdressers [46]. Notably, the association of the hairdressing occupation with cancer and reproductive diseases has been consistently reported [33,45], leading the International Agency for Research on Cancer (IARC) in 2010 to classify the occupational exposure of hairdressers as probably carcinogenic (IARC group 2A).

In summary, the state of the art on occupational exposure to endocrine disruptors in hairdressers highlights the small number of recent studies, the rare reports on the type and magnitude of exposure, the mixture effects of all pollutants, the limitations in establishing the respective contributions of various chemicals, and the causal relationships that do not enable proper assessment of the endocrine-disrupting effects of occupational exposures. Conducting epidemiological studies on this topic is challenging, since endocrine disruptors are ubiquitous compounds, the health effects could be delayed, mixtures need to be considered to assess the endocrine disruption of ingredients in the final product, and a low enough magnitude of exposure, such as dermal exposure to cosmetics, seems not as hazardous as dietary intake [7]. In an attempt to address these gaps in the existing literature and increase current knowledge regarding thyroid-disrupting substances in this understudied population, we propose an exploratory field study in French hairdressers to investigate occupational exposures to the main thyroid disruptors in hair products and their relationship with blood thyroid parameters.

### Study Objectives

**Figure 2.**
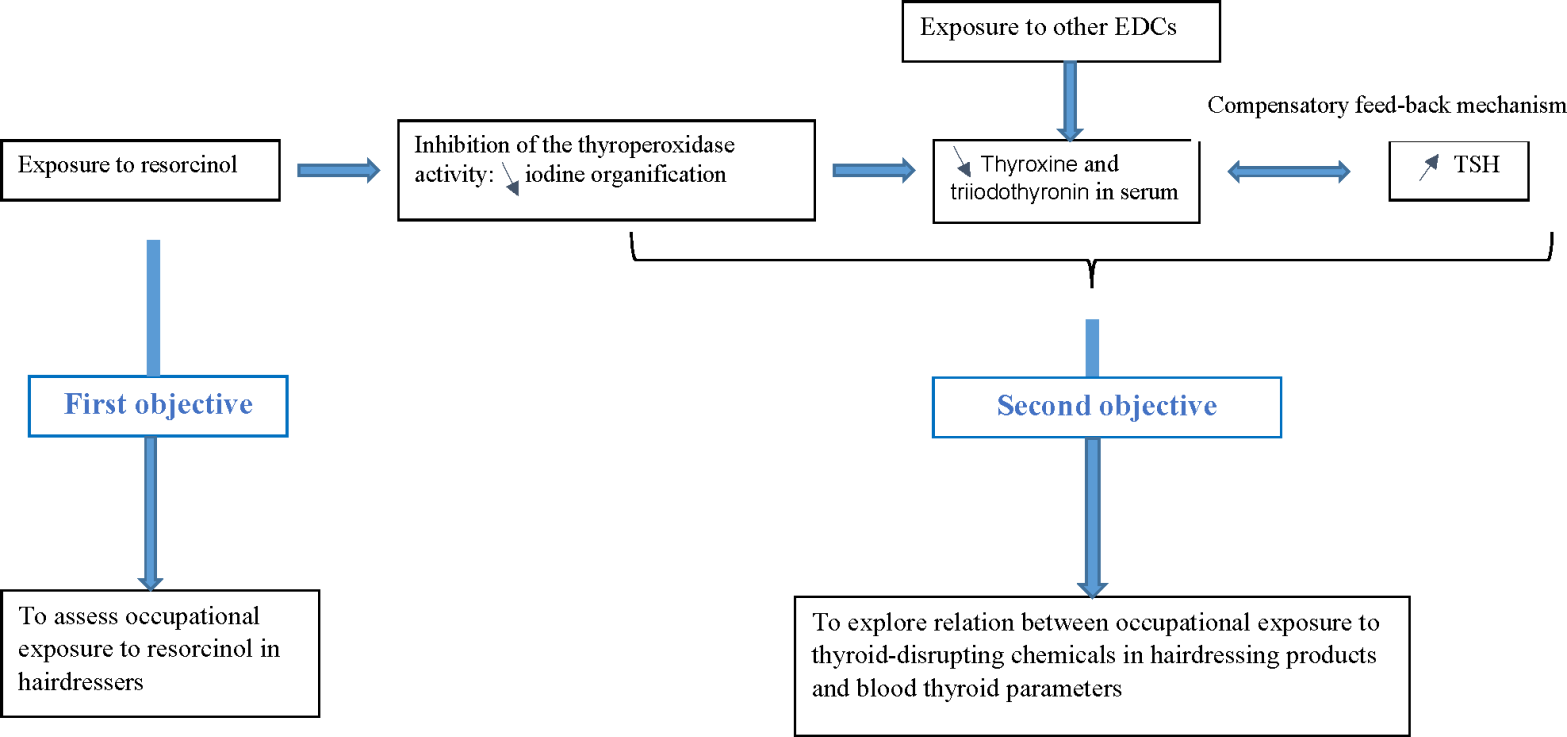
Thyroid-disrupting mode of action of resorcinol and subsequent study objectives. EDC: endocrine-disrupting chemical; TSH: thyroid-stimulating hormone.

Based on the presumed physiopathological mechanism presented in Figure 2, this study aims to address 2 objectives:

- The first objective is to assess occupational exposure to resorcinol, based on urinary measurements in hairdressers working in hair salons.
- The second objective is to explore the association between occupational exposure to resorcinol in hairdressers and biological thyroid parameters, taking into account occupational exposure to other endocrine disruptors, such as parabens and UV filters.

## Methods

### Study Design and Protocol

We designed an occupationally exposed-unexposed cross-sectional study in which hairdressers are followed up during a 5-day working week after at least 1 day of rest. During the working week, urine samples are collected before “day 1” shift (after at least 1 day off), before and after “day 3” and “day 4” shifts, and before “day 5” shift (Multimedia Appendix 1). This sampling strategy relies on the toxicokinetics of resorcinol and the occupational biomonitoring approach tailored for work shifts [40,47].

Few data on resorcinol toxicokinetics are available [29]. In humans, a biological half-life of 4.65 hours was predicted through modeling, and a urinary excretion half-life of 31 hours was reported after percutaneous penetration. For chemicals with short half-lives, within-subject biological concentrations vary strongly over time, and this temporal variability could bias the chemical levels during the toxicologically relevant exposure window [48]. Collecting several samples for each participant and performing intraworker repeated exposure measurements could provide a less biased design by reducing variability in exposure assessment.

Thus, the first sample collected after at least 1 day off work represents the individual background level of the hairdresser, while the following time points of urine collection are based on the anticipated exposure time throughout the work week to properly assess occupational exposure. Finally, we also considered the feasibility of the follow-up within hair salons and designed a sampling strategy that could provide a sufficient number of samples to overcome the issues of within-subject exposure variability. Daily tasks are self-reported by each hairdresser during the working week (daily work sheet), and a visit to the hairdressing salon is conducted by the research team. For unexposed women, only 2 urinary samples are collected: one at the beginning and one at the end of the working week.

At the inclusion medical visit, a blood sample is collected, a medical questionnaire is administered, and a self-administered questionnaire is provided to all participants. At the end of the follow-up, all daily work sheets and the self-administered questionnaire are collected by the research team. The medical questionnaire, self-administered questionnaire, and self-reported daily work sheet are provided in Multimedia Appendix 2 (available only in French). Finally, this protocol was designed to adhere to applicable guidelines for reporting observational studies (STROBE [Strengthening the Reporting of Observational Studies in Epidemiology] statement; Checklist 1) [49]. The completed checklist of items that should be included in reports of observational studies is presented in Multimedia Appendix 2.

### Study Population

#### Inclusion and Exclusion Criteria

The occupationally exposed population consists of female hairdressers aged 18 to 45 years, not being apprentices, working in traditional hairdressing salons, and having a seniority of at least 1 year. Home-dressing and salons with other concomitant activities (nail salon, beauty salon, etc) practiced in the same room are not included.

The occupationally unexposed population (controls) consists of female participants aged 18 to 45 years who are not occupationally exposed to hair products or other chemicals and are employed in office activities, such as secretary, computer specialist, and accounting. Exclusion criteria for both exposed and unexposed groups include a history of thyroid disorders, ongoing or past pregnancy within the last 12 months, current breastfeeding, and a history of severe or chronic diseases. All participants are asked about the date of their last personal hair dyeing (not allowed within 7 days before or during the working week follow-up) and the use of iodine products for medical purposes (imaging or dermal products not allowed within 1 month before inclusion or during the working week follow-up). In case of recent personal hair dyeing or use of iodine products, inclusion should be postponed.

#### Recruitment and Inclusion of Participants

The recruitment of the study population is carried out by occupational health professionals belonging to occupational health centers spread across different French regions. It is important to note that the health monitoring of employees within the occupational health centers is required by law in France, and compulsory medical examinations are regularly scheduled throughout an employee’s working life, starting from the moment of hiring. A specific partnership covering this research was implemented with volunteer health centers. Fourteen occupational health centers (6 from the beginning of the study and 8 additional centers since 2024) volunteered to participate in this study because of their interest in EDCs and work. The recruitment of hairdressers is carried out by the occupational health centers based on a three-step procedure: (1) identification in the medical records of eligible hairdressers who meet the inclusion criteria of age, professional activity, and medical history; (2) identification of the salons where eligible hairdressers are working to obtain verbal approval from the salon manager to participate in the research; and (3) in the case of the manager’s approval, hairdressers are informed about the study and then asked if they are willing to volunteer to participate. Voluntary hairdressers are invited to attend the medical inclusion visit within the occupational health centers. In cases of refusal to participate, nonidentifying data on the reasons for the refusal are collected, if possible, from the salon’s manager and the hairdresser. The recruitment of unexposed participants is carried out during the compulsory medical examination within the occupational health centers, and each eligible woman is invited to participate in the study.

### Ethical Considerations

This study was approved by the French Ethics Committee (Comité de Protection des Personnes Nord Ouest III, reference number: 2022‐88). The processing of the study data was the subject of a declaration to the French Data Protection Authority in September 2021 (Commission Nationale de l’Informatique et des Libertés, registration number 2223653 v 0, September 21, 2021). Participation is voluntary, and each participant is given an information letter to review before volunteering. Each participant provides written, informed consent to participate in the study. The right to privacy and confidentiality of participants is upheld throughout the research. No compensation is provided to participants.

### Data Collection

Once consent is obtained, data collection takes place at different time points—during the inclusion visit within the occupational health center and during a 5-day work week in the hairdressing salon. Data are collected at both individual and salon levels: at the individual level, relating to sociodemographics, medical history, environmental and professional exposure, and blood thyroid parameters; and at the hairdressing salon level, concerning the assessment of exposure throughout a salon visit and the inventory of chemical products.

During the inclusion visit, a medical questionnaire is administered face-to-face by the occupational health professional to collect data about participants’ age, body mass, family history of thyroid disorders, personal use of current medicines, reproductive history, dermal disorders, work characteristics and schedules, and the date of the last personal hair dyeing. Then, a self-administered questionnaire is given to the participant to be completed at home and returned to the study team at the end of the 5-day follow-up. The self-administered questionnaire collects information on sociodemographic factors (educational level and marital status), housing data (address, housing type and age, number of persons, surroundings, etc), tobacco status, current food, water and alcohol consumption, current use of personal care and household products, and career history.

One blood sample is collected at the inclusion visit to measure thyroid hormone–related parameters, including free and total triiodothyronine and thyroxine, TSH, thyroglobulin, thyroperoxidase antibodies, and thyroglobulin antibodies. Because of the circadian rhythms reported in thyroid hormones [50], samples are collected for all participants in the morning during the same time slot (8 AM-12 AM). Serum TSH, free triiodothyronine, and free thyroxine are measured by Access Immunoassay Systems with a DXI Beckman Coulter, Inc, analyzer (reference B63284 Access TSH 3rd IS; reference A13422 Access Free T3; reference 33880 Access Free T4) [51]. Serum thyroglobulin and antibodies to thyroperoxidase and thyroglobulin are measured by chemiluminescence electromagnetic immunoassays with a Cobas e Roche Diagnostics immunoassay analyzer (reference 08906564190 Elecsys Tg II; reference 07026935190 Elecsys Anti-TPO; reference 09005021190 Elecsys Anti-Tg) [52]. Finally, total triiodothyronine and total thyroxine in serum are quantified by a chemiluminescent microparticle immunoassay on the Alinity i analyzer, a recently developed automated immunoassay analyzer [53].

Finally, the protocol for a 5-day work week follow-up is implemented to collect urine samples throughout the week and to gather self-reported data on daily job tasks by means of daily work sheets to be filled out at the workplace.

The exposure assessment is based on a multidisciplinary strategy, including urinary biomonitoring, self-reported work organization and daily job tasks, and a visit to the hair salon, including an inventory of the chemical products.

### Individual Exposure Level

#### Urinary Measures

The urinary biomonitoring relates to the thyroid-disrupting chemicals found in hair products, namely resorcinol, parabens, and UV filters, such as ethylhexyl methoxycinnamate and benzophenone. Considering the issues of toxicokinetics, the different sources and routes of exposure, as well as the individual variability in endocrine disruptor measurements [54], urinary samples are collected at different time points. Hairdressers are asked to collect 6 urine samples during the working week: day 1 preshift, days 3 and 4 preshift and postshift, and day 5 preshift (Multimedia Appendix 1). For controls, 2 urine samples are collected during the working week (day 1 preshift and day 5 preshift). All samples are stored at 4°C for a maximum of 3 hours after the collection time. Each sample is divided into 6 aliquots, which are finally stored at −20 °C until the urinary measurements of the endocrine disruptors are conducted.

Urinary measurements of resorcinol are assessed using a high-performance liquid chromatography-high resolution mass spectrometry method developed by our research team [28]. The lower limit of quantification for resorcinol is 0.3 µg/L, and the method has been fully validated for accuracy, precision, carryover, linearity, recovery, and stability (raw samples and postprocessing stabilities). The urinary measurements of parabens (methyl, ethyl, and propyl-paraben), ethylhexyl methoxycinnamate’s metabolites (4’-methoxyacetophenone, 4-methoxycinnamic acid), and benzophenone-3 will be performed in the same laboratory.

#### Work Organization and Job Tasks

Information on work organization and daily job tasks is collected through questionnaires during the inclusion visit, then during a 5-day working week by means of self-reported daily work sheets. Data on work organization relate to the seniority in hairdressing activity, other concomitant activities, type of employment contract (permanent, fixed-term, temporary), weekly schedules (number of working days, working hours, and consecutive working days), and the date of the last working day. Information on daily job tasks relates to activities during the 5-day follow-up week and includes the number of customers served by the hairdresser and the salon per day, the type and number of hairdressing tasks (shampooing, haircutting, dyeing, bleaching, perming, straightening, styling, modeling), and the type and number of cleaning tasks performed at the workplace (tools, salon, furniture). For each hairdressing and cleaning task, additional questions are asked regarding the use of personal protective equipment (gloves, facial mask), the names of products used, the wearing of wrist or hand or finger jewelry, and smoking during the workday.

### Salon Characteristics and Inventory

A hair salon visit is conducted by 1 to 2 members of the study team in every salon where at least 1 hairdresser is included in the study. The visit starts with an interview with the salon manager, followed by a brief observation of the salon and a complete inventory of hair and chemical products within the salon. Information on work organization (staff, schedules, customers, hairdressing tasks) and salon characteristics (opening year, total surface, number and usage of rooms, customer seats, shampoo bins, ventilation systems, personal protective equipment, cleaning tasks of tools or salon or furniture, care and hair products, cleaning products) is collected. A full inventory of products in the salon is therefore undertaken by means of dedicated numeric tools—bar code scanner devices, mobile phones, and a specific database maintained by our research team. The labels of all hair care and cleaning products are first scanned; then, photos of the name and composition are taken for each product. These data are sent to the database to feed 4 subdatabases. The first subdatabase contains all the products inventoried in the study; the second includes all the products per salon; the third contains all the compounds per product, as mentioned in the label; and the fourth contains a short toxicological information of each compound based on the Chemical Abstracts Service number where available. This includes IARC and CLP classifications for carcinogenic, mutagenic, or toxic for reproduction [55,56] and CLP classification for sensitizers [56], and 3 reference sources for endocrine disruptors. Along with endocrine disruptor lists presented above [6], 2 other sources are consulted: the DEDuCT version 2 (database of EDCs and their toxicity profiles), which compiles information on potential EDCs with supporting evidence from research papers published unto January 2020 [57,58], and a list of substances of interest because of their potential endocrine action published by French Agency for Food, Environmental & Occupational Health & Safety in 2021 [59], based on the DEDuCT database, enriched with more chemicals, such as coformulants in plant protection products (phyto or pesticides) or biocidal products and phyto or biocidal active substances.

### Sample Size and Statistical Methods

The sample size was determined based on the mean serum levels of TSH (2.1 µUI/mL, SD 1.0) and free thyroxine (10.9 ng/L, SD 1.2) in healthy French women aged 35 to 60 years who were included in the Su.Vi.Max study [60]. So far, no data are available to estimate the differences in serum levels between hairdressers and the unexposed group, but we expect fairly small differences for free thyroxine (3%‐4%) and larger ones for TSH levels (13%‐15%).

**Figure 3.**
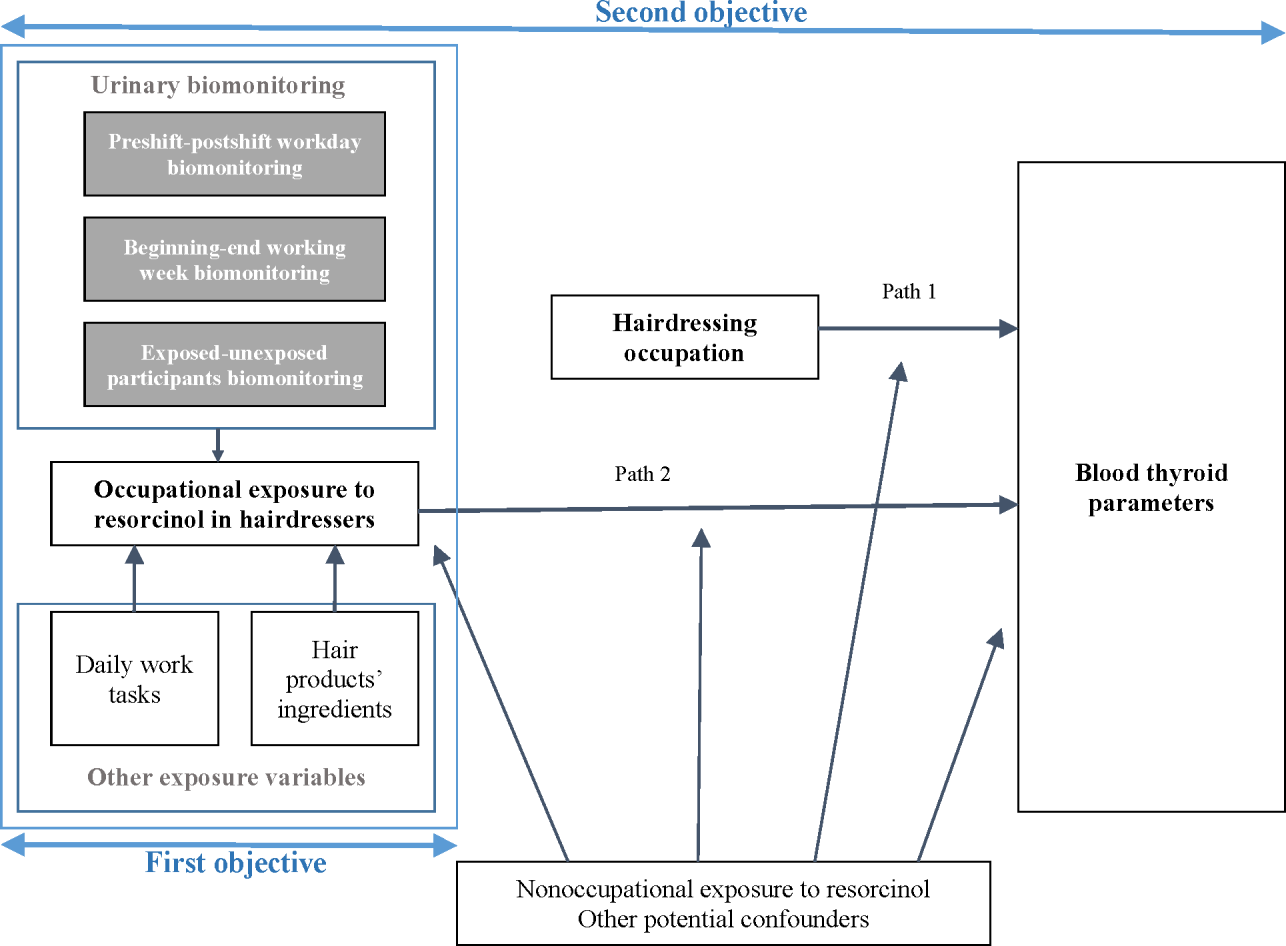
Conceptual framework following the study objectives.

For a ratio of 2 hairdressers to 1 unexposed participant, the population needed to detect these differences between hairdressers and unexposed participants consists of 300 hairdressers and 150 unexposed participants and corresponds to an 80% power at a 5% significance level. Descriptive statistics will be used to describe all population and separately analyze hairdressers and unexposed subjects. To meet the study objectives, different statistical methods will be carried out according to the conceptual framework presented in Figure 3.

For assessing the occupational exposure to resorcinol among hairdressers, urinary concentrations of EDCs at the beginning of the working day will be compared to those at the end of the day, as well as to the concentrations measured at the beginning and end of the working week, taking into account intraindividual and interindividual variability (related to the repetition of samples over 2 days). The mean concentrations will be calculated with all samples collected during the week for exposed and unexposed populations (up to 6 for exposed and 2 for nonoccupationally exposed) and compared between the 2 populations. An in-depth exposure assessment incorporating data from the hairdressing product inventory and daily work sheets will be carried out depending on the quality of the data. Nonoccupational exposures to resorcinol described in the literature will be considered by using data related to personal use of cosmetics, diet, alcohol consumption, and smoking.

For analyzing thyroid-disrupting effects in hairdressers, the relationship with thyroid parameters will be explored using 2 approaches. First, the association between the hairdressing profession and thyroid hormone concentration will be analyzed using linear regression models (path 1), taking into account potential confounding factors collected via questionnaire and linked to participants’ residence, use of personal cosmetic products, consumption of food and drink, smoking, cleaning product use, and occupational history. The different indicators of thyroid function will be treated either independently of each other or simultaneously using multivariate statistical models. Second, the relationship between thyroid parameters and urinary impregnation with resorcinol and other EDCs will be analyzed for all urine samples combined, adjusted for confounders (path 2), allowing us to assess an “average” exposure but also the concentrations measured in the end-of-shift (postshift) samples, under the hypothesis that the latter reflect a “maximum” exposure.

If possible, other individual exposure indices will be determined based on the daily work tasks crossed with the database of salon products and endocrine-disrupting substances. These indices will be used to evaluate occupational exposures and explore associations with thyroid parameters.

All analyses will be performed using Stata software (Stata Corp LP).

## Results

### Anticipated Schedule

The duration of the study spans from early 2022 to the end of 2027. The first year was dedicated to obtaining regulatory committees’ approvals, developing analysis methods for resorcinol and other considered chemicals, preparing for the implementation of the partnership with occupational health centers, achieving data collection tools, and drafting the operating mode for health professionals to follow.

The data collection started in early 2023 and will run until the end of 2026. The final year (2027) will be dedicated to data management, data analysis, and the writing of the study report.

### Update on the Implementation Progress and Participants’ Inclusion

As of December 31, 2025, 66 hairdressers and 30 unexposed participants have been enrolled in the study across 9 occupational health centers. The hairdressers are employed in 54 hair salons where a full inventory of hair and chemical products is also conducted. The implementation of the study in the remaining 5 occupational health centers will continue throughout 2026.

## Discussion

### Expected Findings

This study is the first assessment of occupational exposure to resorcinol in France, a thyroid disruptor that is relevant to human health but not addressed, to the best of our knowledge, in occupational settings. Considering the wide distribution of resorcinol in the environment and the significant increase in the global resorcinol market over the forecast decade, we propose a timely biomonitoring and epidemiological study to analyze its thyroid-disrupting effects related to occupational exposure to hair products.

Part of the “hairdressing exposome,” thyroid disruptors are widely used as ingredients in hair products, and we attempt to take into account, beyond resorcinol, the most common compounds, such as parabens or UV filters.

### Comparison to Prior Work

There is compelling evidence from in vivo*,* in vitro, and epidemiological studies that a growing number of chemicals, at environmentally relevant concentrations, can disrupt the thyroid function and cause adverse health effects.

Recent French epidemiological data showed a decrease in the incidence of acquired hypothyroidism, whereas the incidence of congenital hypothyroidism increased among female participants from 2014 to 2019. However, wide regional disparities within France were highlighted, suggesting a potential impact of environmental factors [61]. The effect of potential environmental factors underlying these disparities was examined with regard to the well-known thyroid disruptors found in outdoor air and tap water [61,62]. In some French regions, prenatal exposure to perchlorate and nitrate ions in tap water was significantly associated with increased neonatal TSH concentrations, and exposure to nitrate ions was associated with an elevated risk of congenital hypothyroidism. Also, prenatal exposure to airborne particulate matter PM_2.5_ and PM_10_ during the third trimester of pregnancy was significantly associated with increased neonatal TSH concentrations. Notably, tap-water monitoring database and outdoor air quality mapping are open-access in France, allowing, where applicable, the integration of these environmental pollutants into the statistical modeling of adverse health effects.

Thyroid-disrupting health effects of exposure to care and other consumer products have been reported [20,21], and their consumption continues today, negatively affecting more susceptible populations, such as pregnant women and their offspring. The neurodevelopmental impact and long-term consequences at a neurocognitive level following in utero exposure to environmental thyroid disruptors were recently reviewed [12]. Massively distributed throughout consumer products, phthalates, bisphenol A, parabens, pesticides, persistent organic pollutants, and perchlorates represent the main maternal exposures to thyroid disruptors, which seem to be associated with long-term consequences, such as a risk for attention or deficit or hyperactivity disorders, autism spectrum disorders, and developmental delay in the offspring.

Regarding occupational exposure to thyroid-disrupting chemicals, most studies have been conducted within specific professional sectors, such as the agricultural and industrial sectors [63], making comparisons between the hairdressing sector and the existing literature limited.

### Impact of the Current Regulation on the Risk Assessment of Endocrine Disruptors

Since there is no specific management of endocrine disruptors in cosmetic products, companies must identify and manage the risks linked to the substances they manufacture and market in the European Union, demonstrate how the substance can be safely used, and communicate the risk management measures to the users [1]. Further, cosmetic products are exempt from the legislation requiring the provision of a safety data sheet.

The identification of endocrine disruptors as SVHC enables the reduction of their use and ultimately their replacement with safer alternatives [64]. Nevertheless, the available regulatory guidelines and toxicological evaluations were not designed to assess endocrine-disrupting properties in themselves and need to be reconsidered to accurately assess thyroid disruption [65]. Relying almost exclusively on animal studies that may not be suitable for assessing thyroid disruption, the existing tools for regulatory evaluations of thyroid disruptors can lead to opposing opinions between regulatory instances, as was the case for resorcinol within the European Union [29]. Thus, the proposal to identify resorcinol as an SVHC was not adopted by EU instances, mostly because of some discrepancies in the animal experimental data required by the regulatory framework for EDC evaluation [65]. Moreover, the reversibility after exposure to clinical hypothyroidism in adults can raise questions, within the regulatory area, about whether such health effects on thyroid function are indeed adverse. It is noteworthy that developmental thyroid disruption can induce long-term changes in neural functions, behavior, and learning and memory in offspring. Therefore, although thyroid markers may return to normal after exposure in adults, some adverse effects may not be reversible in the context of in utero transient developmental exposure.

At the workplace level, the fact that cosmetic products do not require a safety data sheet makes it difficult to track occupational exposure to endocrine-disrupting substances contained in hairdressing products. In an attempt to support occupational health professionals in identifying endocrine disruptors in the workplace, the French Research and Safety Institute for the Prevention of Occupational Accidents and Diseases proposed in 2025 a digital tool for tracking and inventorying endocrine-disrupting chemicals [66].

### Issues of Potential Generalizability and Dissemination Plan

The choice to recruit female participants aged 18 to 45 years having at least 1 year’s seniority in hairdressing and working in traditional hairdressing salons relies on the use of building relatively homogeneous and stable groups concerning both exposure and thyroid parameters. In fact, the majority of hairdressers worldwide are women of childbearing age. Furthermore [1], we can expect that occupational and nonoccupational exposures to cosmetic products differ between young apprentices and experienced hairdressers [2]. The data obtained from the 2007-2008 National Health and Nutrition Examination Survey showed that the relationships between thyroid hormones and endocrine disruptors were different between women and men [67] and between adults and adolescents [68]. Moreover, the prevalence and pattern of thyroid disorders differ between the 2 genders [69], as well as the occupational exposure between traditional hairdressing salons and nail or beauty salons [31]. Finally, although the construction of the study population took into account issues of statistical power and feasibility, the results of the study can serve the whole occupational group, including male hairdressers. Furthermore, considering ethnically similar clientele and comparable type and use of hairdressing products, the results of the study could potentially be generalized beyond France. Also, the urinary sampling framework at different time points throughout a working week (background, pre- and postshift, beginning and end of the week levels) could be generalized to assess occupational exposure to other substances with a short half-time and urinary excretion within 24 hours.

A dissemination plan related to this research has been drawn up under many axes: submission of scientific papers in international peer-reviewed journals related to urinary biomonitoring (analytical development for the quantification of considered endocrine disruptors), the study protocol, and the final results; inventory data dissemination as part of open science (the deposit of dataset in an appropriate external repository) and as scientific papers and communications; written feedback to occupational health center partners (biomonitoring report of proper data and the final report of the study); and French-written scientific papers and communications, as well as the dissemination of the final results to French occupational health and safety professionals.

### Strengths and Limitations

A strength of this study is its multilevel and multidisciplinary approach, including epidemiology, occupational health, biomonitoring, and endocrinology, to set up the study methodology. Furthermore, the collection of exposure data at the hairdresser level and salon level allows for an extensive exposure assessment to thyroid-disrupting chemicals, combining biomonitoring, industrial hygiene, and inventory data exploitation. A specific partnership with occupational health centers is implemented for the recruitment process and data collection, and it is likely that their participation could improve the hairdressers’ participation and the quality of data.

The study has a number of weaknesses. First, our study relies on salon managers’ and hairdressers’ willingness to participate in the study, so that the recruitment is very work-intensive, and it may be difficult to achieve the expected number of participants. To note, the participation rate ranges between 10% and 20% for hair salons and between 40% and 50% for hairdressers belonging to voluntary hair salons. The most common barriers reported by hair salon managers to participation in the study are the work overload, problems with the labor force, lack of interest in the research, concerns about the use of results (ie, hairdressers’ health, job suitability, stock of hairdressing products), study procedures judged as too complicated, and hairdressers’ refusal. Furthermore, noninclusion criteria related to recent reproductive events or thyroid disorders are quite frequently reported by hairdressers.

To improve the participation rate, different tools for better communication with each stakeholder are implemented, such as flyers and posters to inform hairdressers and controls about the study, a newsletter to share information with occupational health centers, and informal discussions with salon managers within the occupational health centers. A second drawback, related to the first, is that this population might not be considered representative of the total French hairdressers. Also, the occupational health centers are volunteers to participate, and we should consider this bias for the issue of representativity as well. Nevertheless, we can point out a wide participation of health centers scattered throughout the French regions. Finally, as this study was not built to deeply collect and analyze environmental pollutants, these factors will not be taken into account in the statistical modeling.

### Conclusions and Perspectives

This research, based on a multidisciplinary approach including biomonitoring and epidemiology, will provide the first insight into occupational exposure to resorcinol in France and will advance our understanding of its thyroid-disrupting effects. Together with the inventory of hair products, these results may strengthen the tools for chemical risk diagnosis and prevention in hairdressing salons. Considering the limitations mentioned above, this study remains exploratory, and the results must be interpreted in the extents of selection bias and statistical power. There is a need for increasing the body of knowledge concerning the hairdressing sector and endocrine-related health effects. Future studies should, among others, be specifically designed to accurately examine cause-effect associations, completely measure hairdressers’ exposome, consider toxicologically relevant mixtures, refine the endocrine-related pathways of adverse outcomes, and broadly adjust for coexisting potential confounders, such as environmental pollutants.

## Supplementary material

10.2196/65833 Multimedia Appendix 1 Study design and data collection time points for exposed and unexposed participants.

10.2196/65833 Multimedia Appendix 2 Main questionnaires used for data collection throughout the study.

10.2196/65833 Checklist 1 STROBE checklist.

## Abbreviations

CLP: classification, labeling, and packaging
EDC: endocrine-disrupting chemical
IARC: International Agency for Research on Cancer
REACH: Registration, Evaluation, Authorization, and Restriction of Chemicals
SCCS: Scientific Committee on Consumer Safety
STROBE: Strengthening the Reporting of Observational Studies in Epidemiology
SVHC: substance of very high concern
TSH: thyroid-stimulating hormone
