# Supplementary material for: Occupational Exposure to Resorcinol and Thyroid-Disrupting Effects: Protocol for an Exploratory Field Study in French Hairdressers
Source: JMIR Res Protoc. 2026 Mar 31;15:e65833. doi: 10.2196/65833 (PMC13037828; doi:10.2196/65833)
Supplement: Multimedia Appendix 1 [file resprot-v15-e65833-s001.docx]

| **Inclusion visit**  **(within 14 days before the**  **follow-up week)** | | **5-day working week follow-up**  **(after at least 24 h off work)** | | | | | | | | | |
| --- | --- | --- | --- | --- | --- | --- | --- | --- | --- | --- | --- |
| **Health assessment** | | **Exposure assessment** | | | | | | | | | |
| Medical questionnaire | **E/U** |  | **Day 1** | | | **Day 2** | **Day 3** | | **Day 4** | | **Day 5** |
| Blood sampling  (8 a.m.-12 a.m.) | **E/U** |  | **Pre-shift** | | **Post-shift** |  | **Pre-shift** | **Post-shift** | **Pre-shift** | **Post-shift** | **Pre-shift** |
| Self-questionnaire | **E/U** |  |  |  |  |  |  |  |  |  |  |
|  | | Daily work sheet | **E** | | | **E** | **E** | | **E** | |  |
|  |  | Urine sampling | **E/U** |  | |  | **E** | **E** | **E** | **E** | **E/U** |
|  |  | Hair salon visit | **E** | | | | | | | | |
