# Supplementary material for: Occupational Exposure to Resorcinol and Thyroid-Disrupting Effects: Protocol for an Exploratory Field Study in French Hairdressers
Source: JMIR Res Protoc. 2026 Mar 31;15:e65833. doi: 10.2196/65833 (PMC13037828; doi:10.2196/65833)
Supplement: Multimedia Appendix 2 [file resprot-v15-e65833-s002.pdf]

Questionnaire visite médicale d'inclusion

Après signature du consentement

N° d'anonymat

Date d'inclusion ☐/☐/☐ *jour mois année*

Quelle est votre taille ? ☐ *cm*

Quel est votre poids ? ☐ *kg*

Date de naissance ☐/☐/☐ *jour mois année*

Pays de naissance ____________

Antécédents familiaux

Un membre de votre famille (père, mère, frère, sœur) a-t-il déjà souffert d'une pathologie de la thyroïde (cancer, hypothyroïdie, hyperthyroïdie, maladie d'Hashimoto, maladie de Graves/Basedow...) ?

○ Oui ○ Non

Traitement médicamenteux

Au cours du dernier mois, avez-vous pris un traitement médicamenteux ?

○ Oui ○ Non

↳ __Si oui__, le(s)quel(s) ?

Utilisez-vous un moyen de contraception ?

○ Oui ○ Non

↳ __Si oui__, quel type de contraception utilisez-vous ?

- ○ Contraception orale (pilule)
- ○ Implant
- ○ Patch
- ○ Injection
- ○ Dispositif intra utérin (stérilet cuivre)
- ○ Dispositif intra utérin (stérilet) hormonal
- ○ Autre : ____________

Vie reproductive

Avez-vous déjà été enceinte ?

○ Oui ○ Non et je n'ai pas essayé ○ Non mais j'ai essayé pendant 12 mois ou plus

↳ __Si oui,__ **Combien de grossesse avez-vous eues au cours de votre vie ?** ☐ *grossesses*

Combien d'accouchements avez-vous eus ? ☐ *accouchement(s)*

À quel âge avez-vous été enceinte pour la première fois ? ☐ *ans*

Combien d'enfants avez-vous eus ? ☐ *enfant(s)* (noter 0 si aucun enfant)

Questionnaire visite médicale d'inclusion

Ménopause

Un médecin vous a-t-il dit que vous étiez ménopausée ?

O Oui O Non

Si oui, à quel âge ? ____ *ans*

Êtes-vous sous traitement substitutif ? O Oui O Non

Si oui, depuis combien de temps ? ____ *mois*

Problèmes cutanés

Avez-vous déjà eu de l'eczéma ?

O Oui O Non

Si oui, en avez-vous actuellement ? O Oui O Non

Avez-vous déjà eu des troubles cutanés autres que de l'eczéma (rougeurs, lésions...) ?

O Oui O Non

Si oui, en avez-vous actuellement ? O Oui O Non

Si oui, le(s)quel(s) ? ____________________

Si oui, en avez-vous sur les mains ou les poignets ?

O Oui O Non

Si oui, avez-vous noté une amélioration pendant les jours de congés ? O Oui O Non

Si oui, avez-vous noté une relation entre ces problèmes cutanés et votre activité professionnelle ? O Oui O Non

Activité professionnelle actuelle

Ne pas compléter les cases ci-dessous

PCS ____

CITP ____

NAF ____

Intitulé du poste occupé actuellement ____________________

Employeur ____________________

Date d'arrivée dans l'entreprise ____ / ____ / ____
jour *mois* *année*

Principales activités exercées ____________________

____

Autre activité professionnelle en dehors de votre employeur principal (exemple : coiffure à domicile...) :

O Oui O Non

Si oui, laquelle(s) : ____________________

____

Questionnaire visite médicale d'inclusion

Quel est actuellement votre contrat de travail ?

○ CDI ○ Intérimaire
○ CDD ○ Autre , lequel ? ______________________

Nombre d'heures travaillées par semaine :

○ Moins de 35 heures ○ 35-39 heures ○ 40 heures ou plus

Nombre d'heures travaillées la semaine dernière :

○ Moins de 35 heures ○ 35-39 heures ○ 40 heures ou plus

Nombre de jours travaillés par semaine : ☐ *jours*

Nombre de journées consécutives travaillées par semaine : ☐ *jours*

Portez-vous des gants de protection lors de vos activités professionnelles ?

○ Jamais ○ Rarement ○ Souvent ○ Toujours ○ Non concernée

Date de la dernière journée de travail ☐ / ☐ / ☐
jour mois année

Usage coloration capillaire

Avez-vous déjà réalisé une coloration, décoloration, mèches ou balayage pour vos propres cheveux (par vous-même ou par un professionnel)

○ Oui ○ Non

➥ **Si oui**, quelle est la date de votre dernière coloration ? ☐ / ☐ / ☐
jour mois année

Si la dernière coloration a eu lieu moins d'une semaine avant le recueil du premier échantillon d'urine, décaler la semaine de suivi de la salariée et le recueil de l'échantillon d'urine.

Avez-vous appliqué vous-même le produit de coloration ?

○ Oui ○ Non

➥ **Si non**, avez-vous réalisé la coloration dans un salon de coiffure ?

○ Oui ○ Non

Les données à caractère personnel communiquées dans le cadre de la recherche sont destinées uniquement au personnel habilité de l'INRS qui est responsable du traitement. Les données sont utilisées uniquement pour permettre de réaliser une étude épidémiologique concernant l'exposition professionnelle des coiffeuses au résorcinol et aux perturbateurs endocriniens et les effets sur la fonction thyroïdienne. L'INRS s'engage à ne pas transmettre ni vendre ces données à un tiers.

En application de la législation en vigueur vous disposez d'un droit d'accès, de rectification et d'effacement de vos données personnelles ainsi que des droits d'opposition au traitement de celles-ci. À ce titre, vous pouvez exercer ces droits en vous adressant au délégué à la protection des données de l'INRS : données.personnelles@inrs.fr. Vous avez également le droit d'introduire une réclamation auprès de la CNIL à l'adresse suivante : https://www.cnil.fr/fr/plaintes.

RESORCINOL AUTO-QUESTIONNAIRE

N° anonymat

Les réponses à ce questionnaires resteront confidentielles et seront traitées de manière globale et anonyme par l'équipe de recherche en charge de cette étude.

Date de remplissage / /
jour *mois* *année*

Caractéristiques individuelles

Situation matrimoniale
- O Mariée
- O Pacsée
- O En union libre
- O Veuve
- O Divorcée
- O Célibataire

Quel est le diplôme le plus élevé que vous ayez obtenu (ou niveau d'études équivalent) ?
- O Aucun diplôme
- O BEPC (brevet des collèges)
- O CAP, BEP
- O Bac ou équivalent
- O Bac +2 ou +3
- O Bac +4
- O Bac +5 ou plus

Lieu d'habitation

Quelle est votre commune de résidence ? ____________________ **Code postal :**

Dans quel type d'habitation résidez-vous actuellement ?
- O Appartement individuel
- O Maison individuelle
- O Résidence collective (foyer, hôtel...)
- O Autre Lequel : ____________

En quelle année a été construit ce logement ?
- O Avant le 1er janvier 1949
- O Après le 1er janvier 1949
- O Je ne sais pas

Depuis combien de temps occupez-vous ce logement ? ☐ *années*

En temps normal, combien d'individus, y compris vous, habitent votre domicile (membres ou non de la famille, peu importe l'âge) ?
- O 1 pers
- O 2 pers
- O 3 pers
- O 4 pers
- O 5 pers ou plus

Le logement que vous occupez actuellement dispose-t-il d'un jardin ? O Oui O Non

Habitez-vous à proximité (<1 km) de :

	Oui	Non
Usine d'incinération	O	O
Centre de recyclage de déchets	O	O
Usine de produits chimiques	O	O
Exploitation agricole (y compris d'élevage) ou d'un champ (y compris vignes, verger ou culture maraichère)	O	O

Tabagisme

Tabac

Avez-vous déjà fumé au moins 100 cigarettes au cours de votre vie ?

O Oui O Non

Si oui, à quel âge avez-vous commencé ? ____ *ans*

Fumez-vous actuellement (au moins 1 cigarette fumée au cours du dernier mois) ?

O Oui O Non

Si non, à quel âge avez vous arrêté ? ____ *ans*

Si oui, combien de cigarettes par mois fumez-vous ? ____ *cigarettes* ____ *paquet(s)*

Consommez-vous d'autres types de tabac que les cigarettes ?

O Oui O Non

Si oui, quel(s) type(s) consommez-vous ?

	Non	Oui	Si oui	nbre / semaine
Cigares	☐	☐	→	
Cigarillos	☐	☐	→	
Pipes	☐	☐	→	
Tabac à mâcher / chiquer	☐	☐	→	

Cigarette électronique

Avez-vous déjà utilisé une cigarette électronique ?

O Oui O Non

Si oui, à quel âge l'avez-vous utilisée pour la première fois ? ____ *ans*

Utilisez-vous actuellement une cigarette électronique ?

O Oui O Non

Si oui, à quelle fréquence l'utilisez-vous ?

O Moins d'un jour par semaine

O Au moins 1 jour par semaine — Nbre de jour / semaine : O 1 jour O 2 jours O 3 jours O 4 jours O 5 jours O 6 jours

O Tous les jours — Nbre d'utilisation / jour : O 1 fois O 2 fois O 3 fois ou plus

Tabagisme passif

Avez-vous déjà vécu avec un(e) ou plusieurs fumeurs(ses) ?

○ Oui ○ Non

➥ **Si oui, vivez-vous actuellement avec un(e) ou plusieurs fumeurs(ses) ?**

○ Oui ○ Non

➥ **Si oui, cette personne fume-t-elle à l'intérieur du domicile ou en votre présence ?**

○ Oui ○ Non

Consommation boissons alcoolisées

Au cours des 12 derniers mois, avez-vous consommé au moins 1 verre d'alcool (vin, bière, cidre, apéritif, digestif...) ?

○ Oui ○ Non

➥ **Si oui, combien de fois en avez-vous consommé en moyenne ?**

○ Moins d'1 jour par mois ○ 1 à 3 jours par mois ○ 1 jour ou plus par semaine ○ Tous les jours

Les jours où vous buvez de l'alcool, combien de verre(s) buvez-vous ?

○ 1 verre ○ 2 verres ○ 3 verres ○ 4 verres ○ 5 verres ou plus

Vous est-il arrivé de boire 4 verres d'alcool ou plus dans une même journée ?

○ Jamais ○ Moins d'1 fois par mois ○ 1 fois par mois ○ 1 fois par semaine ○ Chaque jour ou presque

Alimentation

Habituellement, à quelle fréquence consommez-vous les aliments suivants ?

	Jamais ou presque jamais	< 1 x / semaine	1 x / semaine	2 à 3 x / semaine	4 à 6 x / semaine	1 x ou plus / jour	Si plus d'une fois par jour, nombre de fois par jour
Lait ou produits laitiers (hors fromage)	○	○	○	○	○	○ →	
Fromage	○	○	○	○	○	○ →	
Viande (boeuf, veau, agneau, porc...), volaille (poulet...), lapin	○	○	○	○	○	○ →	
Charcuterie	○	○	○	○	○	○ →	
Fruits de mer ou coquillages (crus ou cuits)	○	○	○	○	○	○ →	
Poissons (crus ou cuits)	○	○	○	○	○	○ →	
Poissons pêchés en rivières (crus ou cuits)	○	○	○	○	○	○ →	
Produits à base de poissons et produits de la mer (poisson pané, terrine ou rillettes de poissons, soupe de poisson...)	○	○	○	○	○	○ →	
Algues séchées ou déshydratées ou produits à bases d'algues	○	○	○	○	○	○ →	
Oeufs	○	○	○	○	○	○ →	
Pains, biscottes, céréales	○	○	○	○	○	○ →	
Pâtes, pommes de terre, riz, semoule	○	○	○	○	○	○ →	
Légumes crus (crudités) ou cuits (haricots verts, ...) sauf féculents (pomme de terre)	○	○	○	○	○	○ →	
Légumes secs (lentilles, haricots blancs, fèves, pois chiches)	○	○	○	○	○	○ →	
Fruits crus ou cuits y compris les jus de fruits (100%)	○	○	○	○	○	○ →	
Plats préparés (industriels, traiteurs)	○	○	○	○	○	○ →	
Soja ou produits à base de soja (y compris boissons au soja)	○	○	○	○	○	○ →	

	Jamais ou presque jamais	< 1 x / semaine	Entre 1 et 3 tasses par mois	1 tasse par semaine	Entre 2 et 5 tasses par semaine	1 tasse par jour ou presque	Plusieurs tasses par jour
Du café	○	○	○	○	○	○	○
Du thé	○	○	○	○	○	○	○

	Jamais ou presque	< 1 x par semaine	Entre 1 et 3 verres / mois	1 verre / semaine	Entre 2 et 5 verres / semaine	1 verre / jour ou presque	2 à 4 verres / jour	4 à 8 verres / jour	Plus de 8 verres / jour
De l'eau du robinet	○	○	○	○	○	○	○	○	○
De l'eau en bouteille ou bonbonne	○	○	○	○	○	○	○	○	○

Avez-vous à votre domicile un dispositif de filtration ou purification d'eau du robinet (pichet filtrant, filtre vissé sur robinet ou dispositif vissé sous l'évier) ?

○ Oui ○ Non ○ Je ne sais pas

Compléments alimentaires

Actuellement, consommez-vous des compléments alimentaires ?

○ Oui ○ Non

Si oui, le(s)quel(s) ? ______________________

Quelle est la date de la dernière prise ? ___ / ___ / ___ (*jour* / *mois* / *année*)

Les compléments alimentaires sont des vitamines, des minéraux, des extraits ou concentrés de plantes, des acides aminés, des protéines, des acides gras essentiels (oméga 3 par exemple), des phyto-oestrogènes, ou tout type de compléments à l'alimentation sous forme de pilules, comprimés, gélules, sachet de poudre, sirop, etc.

Consommation de sel

Chez vous, utilisez-vous principalement (une seule réponse possible) ? :

○ Du sel iodé et fluoré ○ Du sel ni iodé ni fluoré ○ Vous ne savez pas

○ Du sel iodé ○ Vous n'avez pas de sel chez vous

Lorsque vous cuisinez (plats de légumes ou de viandes, cuissons des œufs, préparation des crudités), ajoutez-vous du sel (une seule réponse possible) ?

○ Jamais ○ Rarement ○ Souvent ○ Toujours ○ Vous ne savez pas

Usage de produits cosmétiques

<u>Au cours du dernier mois</u>, avez-vous utilisé personnellement (hors usage professionnel) les produits ci-dessous ?

Produits pour les cheveux

	Jamais	Rarement	1 x / par semaine	Quelques fois / semaine	Tous les jours
Shampoing (liquide, solide, sec)	○	○	○	○	○
Produits coiffants (spray, laque, gel, mousse, cire…)	○	○	○	○	○
Après shampoing	○	○	○	○	○
Colorant, teinture	○	○	○	○	○
Décolorant	○	○	○	○	○
Produits pour permanente	○	○	○	○	○
Défrisant, produit lissant	○	○	○	○	○
Autres produits	○	○	○	○	○

Produits pour le visage

	Jamais	Rarement	1 x / semaine	Quelques fois / semaine	Tous les jours
Savon, gel ou lotion nettoyante, lingettes, exfoliant	○	○	○	○	○
Produits de soins, crème hydratante, crème de nuit, anti-acné, masque…	○	○	○	○	○
Fond de teint (poudre, liquide)	○	○	○	○	○
Démaquillant	○	○	○	○	○
Rouge à lèvres	○	○	○	○	○
Blush, fard à joues	○	○	○	○	○
Produits pour les yeux (mascara, fard à paupières, eyeliner ou crayon pour les yeux)	○	○	○	○	○
Cosmétiques traditionnels (Khôl, Surma, Kajal, Tiro)	○	○	○	○	○
Dentifrice	○	○	○	○	○
Bain de bouche	○	○	○	○	○

Soins pour le corps

	Jamais	Rarement	1 x / semaine	Quelques fois / semaine	Tous les jours
Parfum / Eau de toilette	O	O	O	O	O
Nettoyant pour le corps (solide, liquide, lingettes, exfoliant…)	O	O	O	O	O
Soins pour le corps, les mains ou les pieds (crèmes, laits, huiles, gels…)	O	O	O	O	O
Vernis à ongles	O	O	O	O	O
Dissolvant	O	O	O	O	O
Crèmes solaire (écran solaire)	O	O	O	O	O
Lait ou huile de bronzage	O	O	O	O	O
Crème, lait, huile après-solaire	O	O	O	O	O
Déodorant ou anti-transpirant	O	O	O	O	O
Huiles corporelles	O	O	O	O	O
Crème, cire ou bandes dépilatoires	O	O	O	O	O
Crème, gel, lotion après épilation	O	O	O	O	O
Crème de rasage ou lotions après rasage	O	O	O	O	O

Au cours du dernier mois, êtes-vous allée chez le coiffeur ?

O Oui O Non

Au cours du dernier mois, êtes-vous allée dans un centre de soins esthétiques (massages, soins du visage…) ?

O Oui O Non

Au cours du dernier mois, êtes-vous allée dans un centre de manucure ou de pose de prothèses ongulaires ?

O Oui O Non

Usage de produits ménagers et domestiques

<u>Au cours du mois dernier</u>, avez-vous pratiqué des activités vous ayant conduit à utiliser ou être en contact avec les produits ci-dessous (<u>hors activités professionnelles</u>) ?

	Jamais	Rarement	1 x / semaine	Quelque fois / semaine	Tous les jours
Cire à meuble	○	○	○	○	○
Cire à parquets	○	○	○	○	○
Produits d'entretien (cuisine, salle de bain, toilettes, sols, fenêtres)	○	○	○	○	○
Assouplissant	○	○	○	○	○
Liquide vaisselle	○	○	○	○	○
Vernis à bois	○	○	○	○	○
Peintures	○	○	○	○	○
Solvants	○	○	○	○	○
Cire ou produits d'entretien de la voiture	○	○	○	○	○
Produits pour animaux domestiques (anti-parasitaires, produits de toilettage)	○	○	○	○	○
Pesticides pour l'entretien du jardin, du potager ou sur les plantes d'intérieur	○	○	○	○	○
Nettoyage de vêtements au pressing/laverie (produits nettoyage à sec)	○	○	○	○	○

Carrière professionnelle

Merci de remplir le tableau suivant en commençant par votre emploi <u>le plus récent</u>

Emploi N°1 **(emploi actuel)** De *Mois* / *Année* à *Mois* / *Année*

Employeur et adresse de l'entreprise

Ne pas compléter les cases ci-dessous

PCS

Métier exercé

Principales activités

CITP

NAF

Nombre d'heures travaillées par semaine
- O Moins de 35 h
- O Plus de 40 h
- O De 35 h à 39 h

Horaires de nuit (entre 21h et 7h)
- O Oui
- O Non

Port de gants de protection
- O Jamais
- O Souvent
- O Rarement
- O Toujours

Période sans emploi De *Mois* / *Année* à *Mois* / *Année*

Emploi N°2 De *Mois* / *Année* à *Mois* / *Année*

Ne pas compléter les cases ci-dessous

Employeur et adresse de l'entreprise

PCS

CITP

Métier exercé

Principales activités

NAF

Nombre d'heures travaillées par semaine
- O Moins de 35 h
- O Plus de 40 h
- O De 35 h à 39 h

Horaires de nuit (entre 21h et 7h)
- O Oui
- O Non

Port de gants de protection
- O Jamais
- O Souvent
- O Rarement
- O Toujours

Période sans emploi De *Mois* / *Année* à *Mois* / *Année*

Emploi N°3 De *Mois* / *Année* à *Mois* / *Année*

Ne pas compléter les cases ci-dessous

Employeur et adresse de l'entreprise

PCS

CITP

Métier exercé

Principales activités

NAF

Nombre d'heures travaillées par semaine
- O Moins de 35 h
- O Plus de 40 h
- O De 35 h à 39 h

Horaires de nuit (entre 21h et 7h)
- O Oui
- O Non

Port de gants de protection
- O Jamais
- O Souvent
- O Rarement
- O Toujours

Période sans emploi De *Mois* / *Année* à *Mois* / *Année*

RESORCINOL AUTO-QUESTIONNAIRE

Emploi N°4 De *Mois* / *Année* à *Mois* / *Année*

Ne pas compléter les cases ci-dessous

Employeur et adresse de l'entreprise

PCS

CITP

Métier exercé

Principales activités

NAF

Nombre d'heures travaillées par semaine

- O Moins de 35 h
- O Plus de 40 h
- O De 35 h à 39 h

Horaires de nuit (entre 21h et 7h)

- O Oui
- O Non

Port de gants de protection

- O Jamais
- O Souvent
- O Rarement
- O Toujours

Période sans emploi De *Mois* / *Année* à *Mois* / *Année*

Emploi N°5 De *Mois* / *Année* à *Mois* / *Année*

Ne pas compléter les cases ci-dessous

Employeur et adresse de l'entreprise

PCS

CITP

Métier exercé

Principales activités

NAF

Nombre d'heures travaillées par semaine

- O Moins de 35 h
- O Plus de 40 h
- O De 35 h à 39 h

Horaires de nuit (entre 21h et 7h)

- O Oui
- O Non

Port de gants de protection

- O Jamais
- O Souvent
- O Rarement
- O Toujours

Période sans emploi De *Mois* / *Année* à *Mois* / *Année*

Emploi N°6 De *Mois* / *Année* à *Mois* / *Année*

Ne pas compléter les cases ci-dessous

Employeur et adresse de l'entreprise

PCS

CITP

Métier exercé

Principales activités

NAF

Nombre d'heures travaillées par semaine

- O Moins de 35 h
- O Plus de 40 h
- O De 35 h à 39 h

Horaires de nuit (entre 21h et 7h)

- O Oui
- O Non

Port de gants de protection

- O Jamais
- O Souvent
- O Rarement
- O Toujours

Période sans emploi De *Mois* / *Année* à *Mois* / *Année*

RESORCINOL AUTO-QUESTIONNAIRE

Emploi N°7 De *Mois* / *Année* à *Mois* / *Année*

Ne pas compléter les cases ci-dessous

Employeur adresse de l'entreprise

PCS

CITP

Métier exercé **Principales activités**

NAF

Nombre d'heures travaillées par semaine
- O Moins de 35 h
- O Plus de 40 h
- O De 35 h à 39 h

Horaires de nuit (entre 21h et 7h)
- O Oui O Non

Port de gants de protection
- O Jamais
- O Souvent
- O Rarement
- O Toujours

Période sans emploi De *Mois* / *Année* à *Mois* / *Année*

Emploi N°8 De *Mois* / *Année* à *Mois* / *Année*

Ne pas compléter les cases ci-dessous

Employeur et adresse de l'entreprise

PCS

CITP

Métier exercé **Principales activités**

NAF

Nombre d'heures travaillées par semaine
- O Moins de 35 h
- O Plus de 40 h
- O De 35 h à 39 h

Horaires de nuit (entre 21h et 7h)
- O Oui O Non

Port de gants de protection
- O Jamais
- O Souvent
- O Rarement
- O Toujours

Période sans emploi De *Mois* / *Année* à *Mois* / *Année*

Emploi N°9 De *Mois* / *Année* à *Mois* / *Année*

Ne pas compléter les cases ci-dessous

Employeur et adresse de l'entreprise

PCS

CITP

Métier exercé **Principales activités**

NAF

Nombre d'heures travaillées par semaine
- O Moins de 35 h
- O Plus de 40 h
- O De 35 h à 39 h

Horaires de nuit (entre 21h et 7h)
- O Oui O Non

Port de gants de protection
- O Jamais
- O Souvent
- O Rarement
- O Toujours

Période sans emploi De *Mois* / *Année* à *Mois* / *Année*

RESORCINOL AUTO-QUESTIONNAIRE

Emploi N°10 De *Mois* / *Année* à *Mois* / *Année*

Ne pas compléter les cases ci-dessous

Employeur et adresse de l'entreprise

PCS

CITP

NAF

Métier exercé

Principales activités

Nombre d'heures travaillées par semaine

- O Moins de 35 h
- O Plus de 40 h
- O De 35 h à 39 h

Horaires de nuit (entre 21h et 7h)

- O Oui
- O Non

Port de gants de protection

- O Jamais
- O Souvent
- O Rarement
- O Toujours

Période sans emploi De *Mois* / *Année* à *Mois* / *Année*

Commentaires :

Les données à caractère personnel communiquées dans le cadre de la recherche sont destinées uniquement au personnel habilité de l'INRS qui est responsable du traitement. Les données sont utilisées uniquement pour permettre de réaliser une étude épidémiologique concernant l'exposition professionnelle des coiffeuses au résorcinol et aux perturbateurs endocriniens et les effets sur la fonction thyroïdienne. L'INRS s'engage à ne pas transmettre ni vendre ces données à un tiers.

En application de la législation en vigueur vous disposez d'un droit d'accès, de rectification et d'effacement de vos données personnelles ainsi que des droits d'opposition au traitement de celles-ci. À ce titre, vous pouvez exercer ces droits en vous adressant au délégué à la protection des données de l'INRS : données.personnelles@inrs.fr. Vous avez également le droit d'introduire une réclamation auprès de la CNIL à l'adresse suivante : https://www.cnil.fr/fr/plaintes.

Institut National de Recherche et de Sécurité

Fiches journalières de poste

Ce questionnaire est à remplir tout au long de la semaine, à la fin de votre journée de travail et concerne **uniquement les tâches que vous avez réalisées au cours de la journée**.

Les réponses à ces questions resteront confidentielles et seront traitées de manière globale et anonyme par l'équipe de recherche en charge de cette étude.

Comment les remplir :

- **Pour chaque jour, vous avez 2 pages à remplir : une page avec des <u>questions</u> et une page avec un <u>tableau</u> afin de répertorier les actes effectués dans une journée de travail.**
- **Pour faciliter le remplissage du tableau, un suivi des actes effectués auprès de chaque client est disponible à la suite du tableau : n'hésitez pas à vous en servir.**
- **Si vous n'avez pas réalisé la tâche demandée, indiquez « 0 » dans la colonne « nombre de fois dans la journée » comme illustré dans l'exemple ci-dessous :**

<u>Exemple :</u>

Actes	**Nombre de fois dans la journée**	**Port de gants**			**Port d'un masque**		**Produits utilisés* (nom commercial+ Marque) en LETTRES CAPITALES**
		Oui, gants jetables	Oui gants réutilisables	Non	Oui	Non	
**Shampoing**			X		X		EXTREME LENGHT REDKEN, PRO LONGER L'OREAL
**Coupe**	0						

Jour 1

Date de remplissage : ___/___/_____

Si vous n'avez pas travaillé ce jour, cocher cette case ☐

Heure de début de poste : ___ : ___ Heure de fin de poste : ___ : ___

À quelle heure avez-vous utilisé, pour la première fois dans la journée, un produit capillaire ? ___ :___

À quelle heure avez-vous utilisé, pour la dernière fois dans la journée, un produit capillaire ? ___ :___

Au total, de combien de clients vous êtes-vous occupée aujourd'hui (y compris ceux pour lesquels vous n'avez réalisé que le shampoing) ? _______ *client(s)*

Au total, combien de coiffeur(euses) étaient présent(e)s au salon au cours de la journée ? _______ *coiffeur(euses)*

Au total, combien de clients ont été reçus au salon au cours de la journée (y compris ceux que vous avez pris en charge) ? _______ *client(s)*

Avez-vous porté des bijoux aux mains et/ou poignets au cours de la journée ? ☐ Oui ☐ Non

Avez-vous fumé au cours de la journée ? ☐ Oui ☐ Non

Comment jugez-vous l'activité de cette journée de travail par rapport à une journée de travail « moyenne » ?

☐ Plus faible que la moyenne ☐ Semblable à la moyenne ☐ Plus élevée que la moyenne

Avez- vous des commentaires ou remarques sur cette journée de travail ? (incidents, éclaboussures sur le visage ou la peau, rupture des gants de protection…) ☐ Oui ☐ Non

↳ Si oui, lesquels --

Au cours de la journée de travail, avez-vous réalisé les actes suivants ?

Actes	**Nombre de fois dans la journée**	**Port de gants**			**Port d'un masque**		**Produits utilisés* (nom commercial) en LETTRES CAPITALES**
		Oui, gants jetables	Oui gants réutilisables	Non	Oui	Non	
**Shampoing**							
**Coupe**							
**Coloration**							
Préparation							
Application							
Rinçage							
Nettoyage du bac et ustensiles							
**Décoloration**							
**Coupe après coloration ou décoloration**							
**Permanente**							
**Lissage**							
**Coiffage/Modelage (application gel, cire…)**							
**Nettoyage du matériel (ciseaux, peignes…)**							
**Nettoyage du salon (sol, poste de coiffage…)**							
**Autre :** ____________________							
**Autre :** ____________________							
**Autre :** ____________________							

*Si disponible, se reporter à la liste des produits présents dans votre salon.

Aide au remplissage du tableau précédent

Client 1 :

Tâche 1 : ____________________ **Port de gants** ☐oui ☐ non **Port de masque** ☐oui ☐ non

Produits utilisés : ____

Tâche 2 : ____________________ **Port de gants** ☐oui ☐ non **Port de masque** ☐oui ☐ non

Produits utilisés : ____

Tâche 3 : ____________________ **Port de gants** ☐oui ☐ non **Port de masque** ☐oui ☐ non

Produits utilisés : ____

Tâche 4 : ____________________ **Port de gants** ☐oui ☐ non **Port de masque** ☐oui ☐ non

Produits utilisés : ____

Tâche 5 : ____________________ **Port de gants** ☐oui ☐ non **Port de masque** ☐oui ☐ non

Produits utilisés : ____

Tâche 6 : ____________________ **Port de gants** ☐oui ☐ non **Port de masque** ☐oui ☐ non

Produits utilisés : ____

Remarques :

Client 2 :

Tâche 1 : ____________________ **Port de gants** ☐oui ☐ non **Port de masque** ☐oui ☐ non

Produits utilisés : ____

Tâche 2 : ____________________ **Port de gants** ☐oui ☐ non **Port de masque** ☐oui ☐ non

Produits utilisés : ____

Tâche 3 : ____________________ **Port de gants** ☐oui ☐ non **Port de masque** ☐oui ☐ non

Produits utilisés : ____

Tâche 4 : ____________________ **Port de gants** ☐oui ☐ non **Port de masque** ☐oui ☐ non

Produits utilisés : ____

Tâche 5 : ____________________ **Port de gants** ☐oui ☐ non **Port de masque** ☐oui ☐ non

Produits utilisés : ____

Tâche 6 : ____________________ **Port de gants** ☐oui ☐ non **Port de masque** ☐oui ☐ non

Produits utilisés : ____

Remarques :

Client 3 :

Tâche 1 : .... **Port de gants** ☐oui ☐ non **Port de masque** ☐oui ☐ non

Produits utilisés : ....

Tâche 2 : .... **Port de gants** ☐oui ☐ non **Port de masque** ☐oui ☐ non

Produits utilisés : ....

Tâche 3 : .... **Port de gants** ☐oui ☐ non **Port de masque** ☐oui ☐ non

Produits utilisés : ....

Tâche 4 : .... **Port de gants** ☐oui ☐ non **Port de masque** ☐oui ☐ non

Produits utilisés : ....

Tâche 5 : .... **Port de gants** ☐oui ☐ non **Port de masque** ☐oui ☐ non

Produits utilisés : ....

Tâche 6 : .... **Port de gants** ☐oui ☐ non **Port de masque** ☐oui ☐ non

Produits utilisés : ....

Remarques :

Client 4 :

Tâche 1 : .... **Port de gants** ☐oui ☐ non **Port de masque** ☐oui ☐ non

Produits utilisés : ....

Tâche 2 : .... **Port de gants** ☐oui ☐ non **Port de masque** ☐oui ☐ non

Produits utilisés : ....

Tâche 3 : .... **Port de gants** ☐oui ☐ non **Port de masque** ☐oui ☐ non

Produits utilisés : ....

Tâche 4 : .... **Port de gants** ☐oui ☐ non **Port de masque** ☐oui ☐ non

Produits utilisés : ....

Tâche 5 : .... **Port de gants** ☐oui ☐ non **Port de masque** ☐oui ☐ non

Produits utilisés : ....

Tâche 6 : .... **Port de gants** ☐oui ☐ non **Port de masque** ☐oui ☐ non

Produits utilisés : ....

Remarques :

Client 5 :

Tâche 1 : ____________ **Port de gants** ☐oui ☐ non **Port de masque** ☐oui ☐ non

Produits utilisés : ____________

Tâche 2 : ____________ **Port de gants** ☐oui ☐ non **Port de masque** ☐oui ☐ non

Produits utilisés : ____________

Tâche 3 : ____________ **Port de gants** ☐oui ☐ non **Port de masque** ☐oui ☐ non

Produits utilisés : ____________

Tâche 4 : ____________ **Port de gants** ☐oui ☐ non **Port de masque** ☐oui ☐ non

Produits utilisés : ____________

Tâche 5 : ____________ **Port de gants** ☐oui ☐ non **Port de masque** ☐oui ☐ non

Produits utilisés : ____________

Tâche 6 : ____________ **Port de gants** ☐oui ☐ non **Port de masque** ☐oui ☐ non

Produits utilisés : ____________

Remarques :

Client 6 :

Tâche 1 : ____________ **Port de gants** ☐oui ☐ non **Port de masque** ☐oui ☐ non

Produits utilisés : ____________

Tâche 2 : ____________ **Port de gants** ☐oui ☐ non **Port de masque** ☐oui ☐ non

Produits utilisés : ____________

Tâche 3 : ____________ **Port de gants** ☐oui ☐ non **Port de masque** ☐oui ☐ non

Produits utilisés : ____________

Tâche 4 : ____________ **Port de gants** ☐oui ☐ non **Port de masque** ☐oui ☐ non

Produits utilisés : ____________

Tâche 5 : ____________ **Port de gants** ☐oui ☐ non **Port de masque** ☐oui ☐ non

Produits utilisés : ____________

Tâche 6 : ____________ **Port de gants** ☐oui ☐ non **Port de masque** ☐oui ☐ non

Produits utilisés : ____________

Remarques :

Client 7 :

Tâche 1 : ………………………… **Port de gants** ☐oui ☐ non **Port de masque** ☐oui ☐ non

Produits utilisés : ……………………………………………………………………………………

Tâche 2 : ………………………… **Port de gants** ☐oui ☐ non **Port de masque** ☐oui ☐ non

Produits utilisés : ……………………………………………………………………………………

Tâche 3 : ………………………… **Port de gants** ☐oui ☐ non **Port de masque** ☐oui ☐ non

Produits utilisés : ……………………………………………………………………………………

Tâche 4 : ………………………… **Port de gants** ☐oui ☐ non **Port de masque** ☐oui ☐ non

Produits utilisés : ……………………………………………………………………………………

Tâche 5 : ………………………… **Port de gants** ☐oui ☐ non **Port de masque** ☐oui ☐ non

Produits utilisés : ……………………………………………………………………………………

Tâche 6 : ………………………… **Port de gants** ☐oui ☐ non **Port de masque** ☐oui ☐ non

Produits utilisés : ……………………………………………………………………………………

Remarques :

Client 8 :

Tâche 1 : ………………………… **Port de gants** ☐oui ☐ non **Port de masque** ☐oui ☐ non

Produits utilisés : ……………………………………………………………………………………

Tâche 2 : ………………………… **Port de gants** ☐oui ☐ non **Port de masque** ☐oui ☐ non

Produits utilisés : ……………………………………………………………………………………

Tâche 3 : ………………………… **Port de gants** ☐oui ☐ non **Port de masque** ☐oui ☐ non

Produits utilisés : ……………………………………………………………………………………

Tâche 4 : ………………………… **Port de gants** ☐oui ☐ non **Port de masque** ☐oui ☐ non

Produits utilisés : ……………………………………………………………………………………

Tâche 5 : ………………………… **Port de gants** ☐oui ☐ non **Port de masque** ☐oui ☐ non

Produits utilisés : ……………………………………………………………………………………

Tâche 6 : ………………………… **Port de gants** ☐oui ☐ non **Port de masque** ☐oui ☐ non

Produits utilisés : ……………………………………………………………………………………

Remarques :

Client 9 :

Tâche 1 : ____________ **Port de gants** ☐oui ☐ non **Port de masque** ☐oui ☐ non

Produits utilisés : ____________

Tâche 2 : ____________ **Port de gants** ☐oui ☐ non **Port de masque** ☐oui ☐ non

Produits utilisés : ____________

Tâche 3 : ____________ **Port de gants** ☐oui ☐ non **Port de masque** ☐oui ☐ non

Produits utilisés : ____________

Tâche 4 : ____________ **Port de gants** ☐oui ☐ non **Port de masque** ☐oui ☐ non

Produits utilisés : ____________

Tâche 5 : ____________ **Port de gants** ☐oui ☐ non **Port de masque** ☐oui ☐ non

Produits utilisés : ____________

Tâche 6 : ____________ **Port de gants** ☐oui ☐ non **Port de masque** ☐oui ☐ non

Produits utilisés : ____________

Remarques :

Client 10 :

Tâche 1 : ____________ **Port de gants** ☐oui ☐ non **Port de masque** ☐oui ☐ non

Produits utilisés : ____________

Tâche 2 : ____________ **Port de gants** ☐oui ☐ non **Port de masque** ☐oui ☐ non

Produits utilisés : ____________

Tâche 3 : ____________ **Port de gants** ☐oui ☐ non **Port de masque** ☐oui ☐ non

Produits utilisés : ____________

Tâche 4 : ____________ **Port de gants** ☐oui ☐ non **Port de masque** ☐oui ☐ non

Produits utilisés : ____________

Tâche 5 : ____________ **Port de gants** ☐oui ☐ non **Port de masque** ☐oui ☐ non

Produits utilisés : ____________

Tâche 6 : ____________ **Port de gants** ☐oui ☐ non **Port de masque** ☐oui ☐ non

Produits utilisés : ____________

Remarques :

Jour 2

Date de remplissage : ___/___/_____

Si vous n'avez pas travaillé ce jour, cocher cette case ☐

Heure de début de poste : ___ : ___ Heure de fin de poste : ___ : ___

**À quelle heure avez-vous utilisé, pour la première fois dans la journée, un produit capillaire ?**	____ :____
**À quelle heure avez-vous utilisé, pour la dernière fois dans la journée, un produit capillaire ?**	____ :____
**Au total, de combien de clients vous êtes-vous occupée aujourd'hui (y compris ceux pour lesquels vous n'avez réalisé que le shampoing) ?**	_______ *client(s)*
**Au total, combien de coiffeur(euses) étaient présent(e)s au salon au cours de la journée ?**	_______ *coiffeur(euses)*
**Au total, combien de clients ont été reçus au salon au cours de la journée (y compris ceux que vous avez pris en charge) ?**	_______ *client(s)*
**Avez-vous porté des bijoux aux mains et/ou poignets au cours de la journée ?**	☐ Oui ☐ Non
**Avez-vous fumé au cours de la journée ?**	☐ Oui ☐ Non

Comment jugez-vous l'activité de cette journée de travail par rapport à une journée de travail « moyenne » ?

☐ Plus faible que la moyenne ☐ Semblable à la moyenne ☐ Plus élevée que la moyenne

Avez- vous des commentaires ou remarques sur cette journée de travail ? (incidents, éclaboussures sur le visage ou la peau, rupture des gants de protection…) ☐ Oui ☐ Non

Si oui, lesquels --

Au cours de la journée de travail, avez-vous réalisé les actes suivants ?

Actes	Nombre de fois dans la journée	Port de gants			Port d'un masque		Produits utilisés* (nom commercial) en LETTRES CAPITALES
		Oui, gants jetables	Oui gants réutilisables	Non	Oui	Non	
**Shampoing**							
**Coupe**							
**Coloration**							
Préparation							
Application							
Rinçage							
Nettoyage du bac et ustensiles							
**Décoloration**							
**Coupe après coloration ou décoloration**							
**Permanente**							
**Lissage**							
**Coiffage/Modelage (application gel, cire…)**							
**Nettoyage du matériel (ciseaux, peignes…)**							
**Nettoyage du salon (sol, poste de coiffage…)**							
**Autre : ___________________**							
**Autre : ___________________**							
**Autre : ___________________**							

*Si disponible, se reporter à la liste des produits présents dans votre salon.

Aide au remplissage du tableau précédent

Client 1 :

Tâche 1 : ---------------------- **Port de gants** ☐oui ☐ non **Port de masque** ☐oui ☐ non

Produits utilisés : --

Tâche 2 : ---------------------- **Port de gants** ☐oui ☐ non **Port de masque** ☐oui ☐ non

Produits utilisés : --

Tâche 3 : ---------------------- **Port de gants** ☐oui ☐ non **Port de masque** ☐oui ☐ non

Produits utilisés : --

Tâche 4 : ---------------------- **Port de gants** ☐oui ☐ non **Port de masque** ☐oui ☐ non

Produits utilisés : --

Tâche 5 : ---------------------- **Port de gants** ☐oui ☐ non **Port de masque** ☐oui ☐ non

Produits utilisés : --

Tâche 6 : ---------------------- **Port de gants** ☐oui ☐ non **Port de masque** ☐oui ☐ non

Produits utilisés : --

Remarques :

Client 2 :

Tâche 1 : ---------------------- **Port de gants** ☐oui ☐ non **Port de masque** ☐oui ☐ non

Produits utilisés : --

Tâche 2 : ---------------------- **Port de gants** ☐oui ☐ non **Port de masque** ☐oui ☐ non

Produits utilisés : --

Tâche 3 : ---------------------- **Port de gants** ☐oui ☐ non **Port de masque** ☐oui ☐ non

Produits utilisés : --

Tâche 4 : ---------------------- **Port de gants** ☐oui ☐ non **Port de masque** ☐oui ☐ non

Produits utilisés : --

Tâche 5 : ---------------------- **Port de gants** ☐oui ☐ non **Port de masque** ☐oui ☐ non

Produits utilisés : --

Tâche 6 : ---------------------- **Port de gants** ☐oui ☐ non **Port de masque** ☐oui ☐ non

Produits utilisés : --

Remarques :

Client 3 :

Tâche 1 : .... **Port de gants** ☐oui ☐ non **Port de masque** ☐oui ☐ non

Produits utilisés : ....

Tâche 2 : .... **Port de gants** ☐oui ☐ non **Port de masque** ☐oui ☐ non

Produits utilisés : ....

Tâche 3 : .... **Port de gants** ☐oui ☐ non **Port de masque** ☐oui ☐ non

Produits utilisés : ....

Tâche 4 : .... **Port de gants** ☐oui ☐ non **Port de masque** ☐oui ☐ non

Produits utilisés : ....

Tâche 5 : .... **Port de gants** ☐oui ☐ non **Port de masque** ☐oui ☐ non

Produits utilisés : ....

Tâche 6 : .... **Port de gants** ☐oui ☐ non **Port de masque** ☐oui ☐ non

Produits utilisés : ....

Remarques :

Client 4 :

Tâche 1 : .... **Port de gants** ☐oui ☐ non **Port de masque** ☐oui ☐ non

Produits utilisés : ....

Tâche 2 : .... **Port de gants** ☐oui ☐ non **Port de masque** ☐oui ☐ non

Produits utilisés : ....

Tâche 3 : .... **Port de gants** ☐oui ☐ non **Port de masque** ☐oui ☐ non

Produits utilisés : ....

Tâche 4 : .... **Port de gants** ☐oui ☐ non **Port de masque** ☐oui ☐ non

Produits utilisés : ....

Tâche 5 : .... **Port de gants** ☐oui ☐ non **Port de masque** ☐oui ☐ non

Produits utilisés : ....

Tâche 6 : .... **Port de gants** ☐oui ☐ non **Port de masque** ☐oui ☐ non

Produits utilisés : ....

Remarques :

Client 5 :

Tâche 1 : .... **Port de gants** ☐oui ☐ non **Port de masque** ☐oui ☐ non

Produits utilisés : ....

Tâche 2 : .... **Port de gants** ☐oui ☐ non **Port de masque** ☐oui ☐ non

Produits utilisés : ....

Tâche 3 : .... **Port de gants** ☐oui ☐ non **Port de masque** ☐oui ☐ non

Produits utilisés : ....

Tâche 4 : .... **Port de gants** ☐oui ☐ non **Port de masque** ☐oui ☐ non

Produits utilisés : ....

Tâche 5 : .... **Port de gants** ☐oui ☐ non **Port de masque** ☐oui ☐ non

Produits utilisés : ....

Tâche 6 : .... **Port de gants** ☐oui ☐ non **Port de masque** ☐oui ☐ non

Produits utilisés : ....

Remarques :

Client 6 :

Tâche 1 : .... **Port de gants** ☐oui ☐ non **Port de masque** ☐oui ☐ non

Produits utilisés : ....

Tâche 2 : .... **Port de gants** ☐oui ☐ non **Port de masque** ☐oui ☐ non

Produits utilisés : ....

Tâche 3 : .... **Port de gants** ☐oui ☐ non **Port de masque** ☐oui ☐ non

Produits utilisés : ....

Tâche 4 : .... **Port de gants** ☐oui ☐ non **Port de masque** ☐oui ☐ non

Produits utilisés : ....

Tâche 5 : .... **Port de gants** ☐oui ☐ non **Port de masque** ☐oui ☐ non

Produits utilisés : ....

Tâche 6 : .... **Port de gants** ☐oui ☐ non **Port de masque** ☐oui ☐ non

Produits utilisés : ....

Remarques :

Client 7 :

Tâche 1 : ____________ **Port de gants** ☐oui ☐ non **Port de masque** ☐oui ☐ non

Produits utilisés : ____

Tâche 2 : ____________ **Port de gants** ☐oui ☐ non **Port de masque** ☐oui ☐ non

Produits utilisés : ____

Tâche 3 : ____________ **Port de gants** ☐oui ☐ non **Port de masque** ☐oui ☐ non

Produits utilisés : ____

Tâche 4 : ____________ **Port de gants** ☐oui ☐ non **Port de masque** ☐oui ☐ non

Produits utilisés : ____

Tâche 5 : ____________ **Port de gants** ☐oui ☐ non **Port de masque** ☐oui ☐ non

Produits utilisés : ____

Tâche 6 : ____________ **Port de gants** ☐oui ☐ non **Port de masque** ☐oui ☐ non

Produits utilisés : ____

Remarques :

Client 8 :

Tâche 1 : ____________ **Port de gants** ☐oui ☐ non **Port de masque** ☐oui ☐ non

Produits utilisés : ____

Tâche 2 : ____________ **Port de gants** ☐oui ☐ non **Port de masque** ☐oui ☐ non

Produits utilisés : ____

Tâche 3 : ____________ **Port de gants** ☐oui ☐ non **Port de masque** ☐oui ☐ non

Produits utilisés : ____

Tâche 4 : ____________ **Port de gants** ☐oui ☐ non **Port de masque** ☐oui ☐ non

Produits utilisés : ____

Tâche 5 : ____________ **Port de gants** ☐oui ☐ non **Port de masque** ☐oui ☐ non

Produits utilisés : ____

Tâche 6 : ____________ **Port de gants** ☐oui ☐ non **Port de masque** ☐oui ☐ non

Produits utilisés : ____

Remarques :

Client 9 :

Tâche 1 : ………………… **Port de gants** ☐oui ☐ non **Port de masque** ☐oui ☐ non

Produits utilisés : …………………………………………………………………………

Tâche 2 : ………………… **Port de gants** ☐oui ☐ non **Port de masque** ☐oui ☐ non

Produits utilisés : …………………………………………………………………………

Tâche 3 : ………………… **Port de gants** ☐oui ☐ non **Port de masque** ☐oui ☐ non

Produits utilisés : …………………………………………………………………………

Tâche 4 : ………………… **Port de gants** ☐oui ☐ non **Port de masque** ☐oui ☐ non

Produits utilisés : …………………………………………………………………………

Tâche 5 : ………………… **Port de gants** ☐oui ☐ non **Port de masque** ☐oui ☐ non

Produits utilisés : …………………………………………………………………………

Tâche 6 : ………………… **Port de gants** ☐oui ☐ non **Port de masque** ☐oui ☐ non

Produits utilisés : …………………………………………………………………………

Remarques :

Client 10 :

Tâche 1 : ………………… **Port de gants** ☐oui ☐ non **Port de masque** ☐oui ☐ non

Produits utilisés : …………………………………………………………………………

Tâche 2 : ………………… **Port de gants** ☐oui ☐ non **Port de masque** ☐oui ☐ non

Produits utilisés : …………………………………………………………………………

Tâche 3 : ………………… **Port de gants** ☐oui ☐ non **Port de masque** ☐oui ☐ non

Produits utilisés : …………………………………………………………………………

Tâche 4 : ………………… **Port de gants** ☐oui ☐ non **Port de masque** ☐oui ☐ non

Produits utilisés : …………………………………………………………………………

Tâche 5 : ………………… **Port de gants** ☐oui ☐ non **Port de masque** ☐oui ☐ non

Produits utilisés : …………………………………………………………………………

Tâche 6 : ………………… **Port de gants** ☐oui ☐ non **Port de masque** ☐oui ☐ non

Produits utilisés : …………………………………………………………………………

Remarques :

Jour 3

Date de remplissage : ___/___/_____

Si vous n'avez pas travaillé ce jour, cocher cette case ☐

Heure de début de poste : ___ : ___ Heure de fin de poste : ___ : ___

**À quelle heure avez-vous utilisé, pour la première fois dans la journée, un produit capillaire ?**	___ :___
**À quelle heure avez-vous utilisé, pour la dernière fois dans la journée, un produit capillaire ?**	___ :___
**Au total, de combien de clients vous êtes-vous occupée aujourd'hui (y compris ceux pour lesquels vous n'avez réalisé que le shampoing) ?**	_____ *client(s)*
**Au total, combien de coiffeur(euses) étaient présent(e)s au salon au cours de la journée ?**	_____ *coiffeur(euses)*
**Au total, combien de clients ont été reçus au salon au cours de la journée (y compris ceux que vous avez pris en charge) ?**	_____ *client(s)*
**Avez-vous porté des bijoux aux mains et/ou poignets au cours de la journée ?**	☐ Oui ☐ Non
**Avez-vous fumé au cours de la journée ?**	☐ Oui ☐ Non

Comment jugez-vous l'activité de cette journée de travail par rapport à une journée de travail « moyenne » ?

☐Plus faible que la moyenne ☐ Semblable à la moyenne ☐ Plus élevée que la moyenne

Avez- vous des commentaires ou remarques sur cette journée de travail ? (incidents, éclaboussures sur le visage ou la peau, rupture des gants de protection…) ☐ Oui ☐ Non

Si oui, lesquels --

Au cours de la journée de travail, avez-vous réalisé les actes suivants ?

Actes	**Nombre de fois dans la journée**	**Port de gants**			**Port d'un masque**		**Produits utilisés* (nom commercial) en LETTRES CAPITALES**
		Oui, gants jetables	Oui gants réutilisables	Non	Oui	Non	
**Shampoing**							
**Coupe**							
**Coloration**							
Préparation							
Application							
Rinçage							
Nettoyage du bac et ustensiles							
**Décoloration**							
**Coupe après coloration ou décoloration**							
**Permanente**							
**Lissage**							
**Coiffage/Modelage (application gel, cire…)**							
**Nettoyage du matériel (ciseaux, peignes…)**							
**Nettoyage du salon (sol, poste de coiffage…)**							
**Autre :** ___________________							
**Autre :** ___________________							
**Autre :** ___________________							

*Si disponible, se reporter à la liste des produits présents dans votre salon.

Aide au remplissage du tableau précédent

Client 1 :

Tâche 1 : ………………… **Port de gants** ☐oui ☐ non **Port de masque** ☐oui ☐ non

Produits utilisés : …………………………………………………………

Tâche 2 : ………………… **Port de gants** ☐oui ☐ non **Port de masque** ☐oui ☐ non

Produits utilisés : …………………………………………………………

Tâche 3 : ………………… **Port de gants** ☐oui ☐ non **Port de masque** ☐oui ☐ non

Produits utilisés : …………………………………………………………

Tâche 4 : ………………… **Port de gants** ☐oui ☐ non **Port de masque** ☐oui ☐ non

Produits utilisés : …………………………………………………………

Tâche 5 : ………………… **Port de gants** ☐oui ☐ non **Port de masque** ☐oui ☐ non

Produits utilisés : …………………………………………………………

Tâche 6 : ………………… **Port de gants** ☐oui ☐ non **Port de masque** ☐oui ☐ non

Produits utilisés : …………………………………………………………

Remarques :

Client 2 :

Tâche 1 : ………………… **Port de gants** ☐oui ☐ non **Port de masque** ☐oui ☐ non

Produits utilisés : …………………………………………………………

Tâche 2 : ………………… **Port de gants** ☐oui ☐ non **Port de masque** ☐oui ☐ non

Produits utilisés : …………………………………………………………

Tâche 3 : ………………… **Port de gants** ☐oui ☐ non **Port de masque** ☐oui ☐ non

Produits utilisés : …………………………………………………………

Tâche 4 : ………………… **Port de gants** ☐oui ☐ non **Port de masque** ☐oui ☐ non

Produits utilisés : …………………………………………………………

Tâche 5 : ………………… **Port de gants** ☐oui ☐ non **Port de masque** ☐oui ☐ non

Produits utilisés : …………………………………………………………

Tâche 6 : ………………… **Port de gants** ☐oui ☐ non **Port de masque** ☐oui ☐ non

Produits utilisés : …………………………………………………………

Remarques :

Client 3 :

Tâche 1 : .... **Port de gants** ☐oui ☐ non **Port de masque** ☐oui ☐ non

Produits utilisés : ....

Tâche 2 : .... **Port de gants** ☐oui ☐ non **Port de masque** ☐oui ☐ non

Produits utilisés : ....

Tâche 3 : .... **Port de gants** ☐oui ☐ non **Port de masque** ☐oui ☐ non

Produits utilisés : ....

Tâche 4 : .... **Port de gants** ☐oui ☐ non **Port de masque** ☐oui ☐ non

Produits utilisés : ....

Tâche 5 : .... **Port de gants** ☐oui ☐ non **Port de masque** ☐oui ☐ non

Produits utilisés : ....

Tâche 6 : .... **Port de gants** ☐oui ☐ non **Port de masque** ☐oui ☐ non

Produits utilisés : ....

Remarques :

Client 4 :

Tâche 1 : .... **Port de gants** ☐oui ☐ non **Port de masque** ☐oui ☐ non

Produits utilisés : ....

Tâche 2 : .... **Port de gants** ☐oui ☐ non **Port de masque** ☐oui ☐ non

Produits utilisés : ....

Tâche 3 : .... **Port de gants** ☐oui ☐ non **Port de masque** ☐oui ☐ non

Produits utilisés : ....

Tâche 4 : .... **Port de gants** ☐oui ☐ non **Port de masque** ☐oui ☐ non

Produits utilisés : ....

Tâche 5 : .... **Port de gants** ☐oui ☐ non **Port de masque** ☐oui ☐ non

Produits utilisés : ....

Tâche 6 : .... **Port de gants** ☐oui ☐ non **Port de masque** ☐oui ☐ non

Produits utilisés : ....

Remarques :

Client 5 :

Tâche 1 : .... **Port de gants** ☐oui ☐ non **Port de masque** ☐oui ☐ non

Produits utilisés : ....

Tâche 2 : .... **Port de gants** ☐oui ☐ non **Port de masque** ☐oui ☐ non

Produits utilisés : ....

Tâche 3 : .... **Port de gants** ☐oui ☐ non **Port de masque** ☐oui ☐ non

Produits utilisés : ....

Tâche 4 : .... **Port de gants** ☐oui ☐ non **Port de masque** ☐oui ☐ non

Produits utilisés : ....

Tâche 5 : .... **Port de gants** ☐oui ☐ non **Port de masque** ☐oui ☐ non

Produits utilisés : ....

Tâche 6 : .... **Port de gants** ☐oui ☐ non **Port de masque** ☐oui ☐ non

Produits utilisés : ....

Remarques :

Client 6 :

Tâche 1 : .... **Port de gants** ☐oui ☐ non **Port de masque** ☐oui ☐ non

Produits utilisés : ....

Tâche 2 : .... **Port de gants** ☐oui ☐ non **Port de masque** ☐oui ☐ non

Produits utilisés : ....

Tâche 3 : .... **Port de gants** ☐oui ☐ non **Port de masque** ☐oui ☐ non

Produits utilisés : ....

Tâche 4 : .... **Port de gants** ☐oui ☐ non **Port de masque** ☐oui ☐ non

Produits utilisés : ....

Tâche 5 : .... **Port de gants** ☐oui ☐ non **Port de masque** ☐oui ☐ non

Produits utilisés : ....

Tâche 6 : .... **Port de gants** ☐oui ☐ non **Port de masque** ☐oui ☐ non

Produits utilisés : ....

Remarques :

Client 7 :

Tâche 1 : ....**Port de gants** ☐oui ☐ non **Port de masque** ☐oui ☐ non

Produits utilisés : ....

Tâche 2 : ....**Port de gants** ☐oui ☐ non **Port de masque** ☐oui ☐ non

Produits utilisés : ....

Tâche 3 : ....**Port de gants** ☐oui ☐ non **Port de masque** ☐oui ☐ non

Produits utilisés : ....

Tâche 4 : ....**Port de gants** ☐oui ☐ non **Port de masque** ☐oui ☐ non

Produits utilisés : ....

Tâche 5 : ....**Port de gants** ☐oui ☐ non **Port de masque** ☐oui ☐ non

Produits utilisés : ....

Tâche 6 : ....**Port de gants** ☐oui ☐ non **Port de masque** ☐oui ☐ non

Produits utilisés : ....

Remarques :

Client 8 :

Tâche 1 : ....**Port de gants** ☐oui ☐ non **Port de masque** ☐oui ☐ non

Produits utilisés : ....

Tâche 2 : ....**Port de gants** ☐oui ☐ non **Port de masque** ☐oui ☐ non

Produits utilisés : ....

Tâche 3 : ....**Port de gants** ☐oui ☐ non **Port de masque** ☐oui ☐ non

Produits utilisés : ....

Tâche 4 : ....**Port de gants** ☐oui ☐ non **Port de masque** ☐oui ☐ non

Produits utilisés : ....

Tâche 5 : ....**Port de gants** ☐oui ☐ non **Port de masque** ☐oui ☐ non

Produits utilisés : ....

Tâche 6 : ....**Port de gants** ☐oui ☐ non **Port de masque** ☐oui ☐ non

Produits utilisés : ....

Remarques :

Client 9 :

Tâche 1 : .... **Port de gants** ☐oui ☐ non **Port de masque** ☐oui ☐ non

Produits utilisés : ....

Tâche 2 : .... **Port de gants** ☐oui ☐ non **Port de masque** ☐oui ☐ non

Produits utilisés : ....

Tâche 3 : .... **Port de gants** ☐oui ☐ non **Port de masque** ☐oui ☐ non

Produits utilisés : ....

Tâche 4 : .... **Port de gants** ☐oui ☐ non **Port de masque** ☐oui ☐ non

Produits utilisés : ....

Tâche 5 : .... **Port de gants** ☐oui ☐ non **Port de masque** ☐oui ☐ non

Produits utilisés : ....

Tâche 6 : .... **Port de gants** ☐oui ☐ non **Port de masque** ☐oui ☐ non

Produits utilisés : ....

Remarques :

Client 10 :

Tâche 1 : .... **Port de gants** ☐oui ☐ non **Port de masque** ☐oui ☐ non

Produits utilisés : ....

Tâche 2 : .... **Port de gants** ☐oui ☐ non **Port de masque** ☐oui ☐ non

Produits utilisés : ....

Tâche 3 : .... **Port de gants** ☐oui ☐ non **Port de masque** ☐oui ☐ non

Produits utilisés : ....

Tâche 4 : .... **Port de gants** ☐oui ☐ non **Port de masque** ☐oui ☐ non

Produits utilisés : ....

Tâche 5 : .... **Port de gants** ☐oui ☐ non **Port de masque** ☐oui ☐ non

Produits utilisés : ....

Tâche 6 : .... **Port de gants** ☐oui ☐ non **Port de masque** ☐oui ☐ non

Produits utilisés : ....

Remarques :

Jour 4

Date de remplissage : ___/___/_____

Si vous n'avez pas travaillé ce jour, cocher cette case ☐

Heure de début de poste : ___ : ___ Heure de fin de poste : ___ : ___

**À quelle heure avez-vous utilisé, pour la première fois dans la journée, un produit capillaire ?**	___ : ___
**À quelle heure avez-vous utilisé, pour la dernière fois dans la journée, un produit capillaire ?**	___ : ___
**Au total, de combien de clients vous êtes-vous occupée aujourd'hui (y compris ceux pour lesquels vous n'avez réalisé que le shampoing) ?**	_____ *client(s)*
**Au total, combien de coiffeur(euses) étaient présent(e)s au salon au cours de la journée ?**	_____ *coiffeur(euses)*
**Au total, combien de clients ont été reçus au salon au cours de la journée (y compris ceux que vous avez pris en charge) ?**	_____ *client(s)*
**Avez-vous porté des bijoux aux mains et/ou poignets au cours de la journée ?**	☐ Oui ☐ Non
**Avez-vous fumé au cours de la journée ?**	☐ Oui ☐ Non

Comment jugez-vous l'activité de cette journée de travail par rapport à une journée de travail « moyenne » ?

☐Plus faible que la moyenne ☐ Semblable à la moyenne ☐ Plus élevée que la moyenne

Avez- vous des commentaires ou remarques sur cette journée de travail ? (incidents, éclaboussures sur le visage ou la peau, rupture des gants de protection…) ☐ Oui ☐ Non

Si oui, lesquels --

Au cours de la journée de travail, avez-vous réalisé les actes suivants ?

Actes	**Nombre de fois dans la journée**	**Port de gants**			**Port d'un masque**		**Produits utilisés* (nom commercial) en LETTRES CAPITALES**
		Oui, gants jetables	Oui gants réutilisables	Non	Oui	Non	
**Shampoing**							
**Coupe**							
**Coloration**							
Préparation							
Application							
Rinçage							
Nettoyage du bac et ustensiles							
**Décoloration**							
**Coupe après coloration ou décoloration**							
**Permanente**							
**Lissage**							
**Coiffage/Modelage (application gel, cire…)**							
**Nettoyage du matériel (ciseaux, peignes…)**							
**Nettoyage du salon (sol, poste de coiffage…)**							
**Autre :** ____________________							
**Autre :** ____________________							
**Autre :** ____________________							

*Si disponible, se reporter à la liste des produits présents dans votre salon.

Aide au remplissage du tableau précédent

Client 1 :

Tâche 1 : ------------------ **Port de gants** ☐oui ☐ non **Port de masque** ☐oui ☐ non

Produits utilisés : --

Tâche 2 : ------------------ **Port de gants** ☐oui ☐ non **Port de masque** ☐oui ☐ non

Produits utilisés : --

Tâche 3 : ------------------ **Port de gants** ☐oui ☐ non **Port de masque** ☐oui ☐ non

Produits utilisés : --

Tâche 4 : ------------------ **Port de gants** ☐oui ☐ non **Port de masque** ☐oui ☐ non

Produits utilisés : --

Tâche 5 : ------------------ **Port de gants** ☐oui ☐ non **Port de masque** ☐oui ☐ non

Produits utilisés : --

Tâche 6 : ------------------ **Port de gants** ☐oui ☐ non **Port de masque** ☐oui ☐ non

Produits utilisés : --

Remarques :

Client 2 :

Tâche 1 : ------------------ **Port de gants** ☐oui ☐ non **Port de masque** ☐oui ☐ non

Produits utilisés : --

Tâche 2 : ------------------ **Port de gants** ☐oui ☐ non **Port de masque** ☐oui ☐ non

Produits utilisés : --

Tâche 3 : ------------------ **Port de gants** ☐oui ☐ non **Port de masque** ☐oui ☐ non

Produits utilisés : --

Tâche 4 : ------------------ **Port de gants** ☐oui ☐ non **Port de masque** ☐oui ☐ non

Produits utilisés : --

Tâche 5 : ------------------ **Port de gants** ☐oui ☐ non **Port de masque** ☐oui ☐ non

Produits utilisés : --

Tâche 6 : ------------------ **Port de gants** ☐oui ☐ non **Port de masque** ☐oui ☐ non

Produits utilisés : --

Remarques :

Client 3 :

Tâche 1 : ---------------------- **Port de gants** ☐oui ☐ non **Port de masque** ☐oui ☐ non

Produits utilisés : --

Tâche 2 : ---------------------- **Port de gants** ☐oui ☐ non **Port de masque** ☐oui ☐ non

Produits utilisés : --

Tâche 3 : ---------------------- **Port de gants** ☐oui ☐ non **Port de masque** ☐oui ☐ non

Produits utilisés : --

Tâche 4 : ---------------------- **Port de gants** ☐oui ☐ non **Port de masque** ☐oui ☐ non

Produits utilisés : --

Tâche 5 : ---------------------- **Port de gants** ☐oui ☐ non **Port de masque** ☐oui ☐ non

Produits utilisés : --

Tâche 6 : ---------------------- **Port de gants** ☐oui ☐ non **Port de masque** ☐oui ☐ non

Produits utilisés : --

Remarques :

Client 4 :

Tâche 1 : ---------------------- **Port de gants** ☐oui ☐ non **Port de masque** ☐oui ☐ non

Produits utilisés : --

Tâche 2 : ---------------------- **Port de gants** ☐oui ☐ non **Port de masque** ☐oui ☐ non

Produits utilisés : --

Tâche 3 : ---------------------- **Port de gants** ☐oui ☐ non **Port de masque** ☐oui ☐ non

Produits utilisés : --

Tâche 4 : ---------------------- **Port de gants** ☐oui ☐ non **Port de masque** ☐oui ☐ non

Produits utilisés : --

Tâche 5 : ---------------------- **Port de gants** ☐oui ☐ non **Port de masque** ☐oui ☐ non

Produits utilisés : --

Tâche 6 : ---------------------- **Port de gants** ☐oui ☐ non **Port de masque** ☐oui ☐ non

Produits utilisés : --

Remarques :

Client 5 :

Tâche 1 : .... **Port de gants** ☐oui ☐ non **Port de masque** ☐oui ☐ non

Produits utilisés : ....

Tâche 2 : .... **Port de gants** ☐oui ☐ non **Port de masque** ☐oui ☐ non

Produits utilisés : ....

Tâche 3 : .... **Port de gants** ☐oui ☐ non **Port de masque** ☐oui ☐ non

Produits utilisés : ....

Tâche 4 : .... **Port de gants** ☐oui ☐ non **Port de masque** ☐oui ☐ non

Produits utilisés : ....

Tâche 5 : .... **Port de gants** ☐oui ☐ non **Port de masque** ☐oui ☐ non

Produits utilisés : ....

Tâche 6 : .... **Port de gants** ☐oui ☐ non **Port de masque** ☐oui ☐ non

Produits utilisés : ....

Remarques :

Client 6 :

Tâche 1 : .... **Port de gants** ☐oui ☐ non **Port de masque** ☐oui ☐ non

Produits utilisés : ....

Tâche 2 : .... **Port de gants** ☐oui ☐ non **Port de masque** ☐oui ☐ non

Produits utilisés : ....

Tâche 3 : .... **Port de gants** ☐oui ☐ non **Port de masque** ☐oui ☐ non

Produits utilisés : ....

Tâche 4 : .... **Port de gants** ☐oui ☐ non **Port de masque** ☐oui ☐ non

Produits utilisés : ....

Tâche 5 : .... **Port de gants** ☐oui ☐ non **Port de masque** ☐oui ☐ non

Produits utilisés : ....

Tâche 6 : .... **Port de gants** ☐oui ☐ non **Port de masque** ☐oui ☐ non

Produits utilisés : ....

Remarques :

Client 7 :

Tâche 1 : ………………………… **Port de gants** ☐oui ☐ non **Port de masque** ☐oui ☐ non

Produits utilisés : ……………………………………………………………………………………

Tâche 2 : ………………………… **Port de gants** ☐oui ☐ non **Port de masque** ☐oui ☐ non

Produits utilisés : ……………………………………………………………………………………

Tâche 3 : ………………………… **Port de gants** ☐oui ☐ non **Port de masque** ☐oui ☐ non

Produits utilisés : ……………………………………………………………………………………

Tâche 4 : ………………………… **Port de gants** ☐oui ☐ non **Port de masque** ☐oui ☐ non

Produits utilisés : ……………………………………………………………………………………

Tâche 5 : ………………………… **Port de gants** ☐oui ☐ non **Port de masque** ☐oui ☐ non

Produits utilisés : ……………………………………………………………………………………

Tâche 6 : ………………………… **Port de gants** ☐oui ☐ non **Port de masque** ☐oui ☐ non

Produits utilisés : ……………………………………………………………………………………

Remarques :

Client 8 :

Tâche 1 : ………………………… **Port de gants** ☐oui ☐ non **Port de masque** ☐oui ☐ non

Produits utilisés : ……………………………………………………………………………………

Tâche 2 : ………………………… **Port de gants** ☐oui ☐ non **Port de masque** ☐oui ☐ non

Produits utilisés : ……………………………………………………………………………………

Tâche 3 : ………………………… **Port de gants** ☐oui ☐ non **Port de masque** ☐oui ☐ non

Produits utilisés : ……………………………………………………………………………………

Tâche 4 : ………………………… **Port de gants** ☐oui ☐ non **Port de masque** ☐oui ☐ non

Produits utilisés : ……………………………………………………………………………………

Tâche 5 : ………………………… **Port de gants** ☐oui ☐ non **Port de masque** ☐oui ☐ non

Produits utilisés : ……………………………………………………………………………………

Tâche 6 : ………………………… **Port de gants** ☐oui ☐ non **Port de masque** ☐oui ☐ non

Produits utilisés : ……………………………………………………………………………………

Remarques :

Client 9 :

Tâche 1 : ………………… **Port de gants** ☐oui ☐ non **Port de masque** ☐oui ☐ non

Produits utilisés : …………………………………………………………………………

Tâche 2 : ………………… **Port de gants** ☐oui ☐ non **Port de masque** ☐oui ☐ non

Produits utilisés : …………………………………………………………………………

Tâche 3 : ………………… **Port de gants** ☐oui ☐ non **Port de masque** ☐oui ☐ non

Produits utilisés : …………………………………………………………………………

Tâche 4 : ………………… **Port de gants** ☐oui ☐ non **Port de masque** ☐oui ☐ non

Produits utilisés : …………………………………………………………………………

Tâche 5 : ………………… **Port de gants** ☐oui ☐ non **Port de masque** ☐oui ☐ non

Produits utilisés : …………………………………………………………………………

Tâche 6 : ………………… **Port de gants** ☐oui ☐ non **Port de masque** ☐oui ☐ non

Produits utilisés : …………………………………………………………………………

Remarques :

Client 10 :

Tâche 1 : ………………… **Port de gants** ☐oui ☐ non **Port de masque** ☐oui ☐ non

Produits utilisés : …………………………………………………………………………

Tâche 2 : ………………… **Port de gants** ☐oui ☐ non **Port de masque** ☐oui ☐ non

Produits utilisés : …………………………………………………………………………

Tâche 3 : ………………… **Port de gants** ☐oui ☐ non **Port de masque** ☐oui ☐ non

Produits utilisés : …………………………………………………………………………

Tâche 4 : ………………… **Port de gants** ☐oui ☐ non **Port de masque** ☐oui ☐ non

Produits utilisés : …………………………………………………………………………

Tâche 5 : ………………… **Port de gants** ☐oui ☐ non **Port de masque** ☐oui ☐ non

Produits utilisés : …………………………………………………………………………

Tâche 6 : ………………… **Port de gants** ☐oui ☐ non **Port de masque** ☐oui ☐ non

Produits utilisés : …………………………………………………………………………

Remarques :

Jour 5

Date de remplissage : ___/___/_____

Si vous n'avez pas travaillé ce jour, cocher cette case ☐

Heure de début de poste : ___ : ___ Heure de fin de poste : ___ : ___

À quelle heure avez-vous utilisé, <u>pour la première fois</u> dans la journée, un produit capillaire ? ____ :____

À quelle heure avez-vous utilisé, <u>pour la dernière fois</u> dans la journée, un produit capillaire ? ____ :____

Au total, de combien de clients vous êtes-vous occupée aujourd'hui (y compris ceux pour lesquels vous n'avez réalisé que le shampoing) ? _______ *client(s)*

Au total, combien de coiffeur(euses) étaient présent(e)s au salon au cours de la journée ? _______ *coiffeur(euses)*

Au total, combien de clients ont été reçus au salon au cours de la journée (y compris ceux que vous avez pris en charge) ? _______ *client(s)*

Avez-vous porté des bijoux aux mains et/ou poignets au cours de la journée ? ☐ Oui ☐ Non

Avez-vous fumé au cours de la journée ? ☐ Oui ☐ Non

Comment jugez-vous l'activité de cette journée de travail par rapport à une journée de travail « moyenne » ?

☐ Plus faible que la moyenne ☐ Semblable à la moyenne ☐ Plus élevée que la moyenne

Avez- vous des commentaires ou remarques sur cette journée de travail ? (incidents, éclaboussures sur le visage ou la peau, rupture des gants de protection…) ☐ Oui ☐ Non

↳ <u>Si oui</u>, lesquels --

Au cours de la journée de travail, avez-vous réalisé les actes suivants ?

Actes	**Nombre de fois dans la journée**	**Port de gants**			**Port d'un masque**		**Produits utilisés* (nom commercial) en LETTRES CAPITALES**
		Oui, gants jetables	Oui gants réutilisables	Non	Oui	Non	
**Shampoing**							
**Coupe**							
**Coloration**							
Préparation							
Application							
Rinçage							
Nettoyage du bac et ustensiles							
**Décoloration**							
**Coupe après coloration ou décoloration**							
**Permanente**							
**Lissage**							
**Coiffage/Modelage (application gel, cire…)**							
**Nettoyage du matériel (ciseaux, peignes…)**							
**Nettoyage du salon (sol, poste de coiffage…)**							
**Autre :** ____________________							
**Autre :** ____________________							
**Autre :** ____________________							

*Si disponible, se reporter à la liste des produits présents dans votre salon.

Aide au remplissage du tableau précédent

Client 1 :

Tâche 1 : ____________________ **Port de gants** ☐oui ☐ non **Port de masque** ☐oui ☐ non

Produits utilisés : ____

Tâche 2 : ____________________ **Port de gants** ☐oui ☐ non **Port de masque** ☐oui ☐ non

Produits utilisés : ____

Tâche 3 : ____________________ **Port de gants** ☐oui ☐ non **Port de masque** ☐oui ☐ non

Produits utilisés : ____

Tâche 4 : ____________________ **Port de gants** ☐oui ☐ non **Port de masque** ☐oui ☐ non

Produits utilisés : ____

Tâche 5 : ____________________ **Port de gants** ☐oui ☐ non **Port de masque** ☐oui ☐ non

Produits utilisés : ____

Tâche 6 : ____________________ **Port de gants** ☐oui ☐ non **Port de masque** ☐oui ☐ non

Produits utilisés : ____

Remarques :

Client 2 :

Tâche 1 : ____________________ **Port de gants** ☐oui ☐ non **Port de masque** ☐oui ☐ non

Produits utilisés : ____

Tâche 2 : ____________________ **Port de gants** ☐oui ☐ non **Port de masque** ☐oui ☐ non

Produits utilisés : ____

Tâche 3 : ____________________ **Port de gants** ☐oui ☐ non **Port de masque** ☐oui ☐ non

Produits utilisés : ____

Tâche 4 : ____________________ **Port de gants** ☐oui ☐ non **Port de masque** ☐oui ☐ non

Produits utilisés : ____

Tâche 5 : ____________________ **Port de gants** ☐oui ☐ non **Port de masque** ☐oui ☐ non

Produits utilisés : ____

Tâche 6 : ____________________ **Port de gants** ☐oui ☐ non **Port de masque** ☐oui ☐ non

Produits utilisés : ____

Remarques :

Client 3 :

Tâche 1 : ____________________ **Port de gants** ☐oui ☐ non **Port de masque** ☐oui ☐ non

Produits utilisés : --

Tâche 2 : ____________________ **Port de gants** ☐oui ☐ non **Port de masque** ☐oui ☐ non

Produits utilisés : --

Tâche 3 : ____________________ **Port de gants** ☐oui ☐ non **Port de masque** ☐oui ☐ non

Produits utilisés : --

Tâche 4 : ____________________ **Port de gants** ☐oui ☐ non **Port de masque** ☐oui ☐ non

Produits utilisés : --

Tâche 5 : ____________________ **Port de gants** ☐oui ☐ non **Port de masque** ☐oui ☐ non

Produits utilisés : --

Tâche 6 : ____________________ **Port de gants** ☐oui ☐ non **Port de masque** ☐oui ☐ non

Produits utilisés : --

Remarques :

Client 4 :

Tâche 1 : ____________________ **Port de gants** ☐oui ☐ non **Port de masque** ☐oui ☐ non

Produits utilisés : --

Tâche 2 : ____________________ **Port de gants** ☐oui ☐ non **Port de masque** ☐oui ☐ non

Produits utilisés : --

Tâche 3 : ____________________ **Port de gants** ☐oui ☐ non **Port de masque** ☐oui ☐ non

Produits utilisés : --

Tâche 4 : ____________________ **Port de gants** ☐oui ☐ non **Port de masque** ☐oui ☐ non

Produits utilisés : --

Tâche 5 : ____________________ **Port de gants** ☐oui ☐ non **Port de masque** ☐oui ☐ non

Produits utilisés : --

Tâche 6 : ____________________ **Port de gants** ☐oui ☐ non **Port de masque** ☐oui ☐ non

Produits utilisés : --

Remarques :

Client 5 :

Tâche 1 : .... **Port de gants** ☐oui ☐ non **Port de masque** ☐oui ☐ non

Produits utilisés : ....

Tâche 2 : .... **Port de gants** ☐oui ☐ non **Port de masque** ☐oui ☐ non

Produits utilisés : ....

Tâche 3 : .... **Port de gants** ☐oui ☐ non **Port de masque** ☐oui ☐ non

Produits utilisés : ....

Tâche 4 : .... **Port de gants** ☐oui ☐ non **Port de masque** ☐oui ☐ non

Produits utilisés : ....

Tâche 5 : .... **Port de gants** ☐oui ☐ non **Port de masque** ☐oui ☐ non

Produits utilisés : ....

Tâche 6 : .... **Port de gants** ☐oui ☐ non **Port de masque** ☐oui ☐ non

Produits utilisés : ....

Remarques :

Client 6 :

Tâche 1 : .... **Port de gants** ☐oui ☐ non **Port de masque** ☐oui ☐ non

Produits utilisés : ....

Tâche 2 : .... **Port de gants** ☐oui ☐ non **Port de masque** ☐oui ☐ non

Produits utilisés : ....

Tâche 3 : .... **Port de gants** ☐oui ☐ non **Port de masque** ☐oui ☐ non

Produits utilisés : ....

Tâche 4 : .... **Port de gants** ☐oui ☐ non **Port de masque** ☐oui ☐ non

Produits utilisés : ....

Tâche 5 : .... **Port de gants** ☐oui ☐ non **Port de masque** ☐oui ☐ non

Produits utilisés : ....

Tâche 6 : .... **Port de gants** ☐oui ☐ non **Port de masque** ☐oui ☐ non

Produits utilisés : ....

Remarques :

Client 7 :

Tâche 1 : ____________________ **Port de gants** ☐oui ☐ non **Port de masque** ☐oui ☐ non

Produits utilisés : ____

Tâche 2 : ____________________ **Port de gants** ☐oui ☐ non **Port de masque** ☐oui ☐ non

Produits utilisés : ____

Tâche 3 : ____________________ **Port de gants** ☐oui ☐ non **Port de masque** ☐oui ☐ non

Produits utilisés : ____

Tâche 4 : ____________________ **Port de gants** ☐oui ☐ non **Port de masque** ☐oui ☐ non

Produits utilisés : ____

Tâche 5 : ____________________ **Port de gants** ☐oui ☐ non **Port de masque** ☐oui ☐ non

Produits utilisés : ____

Tâche 6 : ____________________ **Port de gants** ☐oui ☐ non **Port de masque** ☐oui ☐ non

Produits utilisés : ____

Remarques :

Client 8 :

Tâche 1 : ____________________ **Port de gants** ☐oui ☐ non **Port de masque** ☐oui ☐ non

Produits utilisés : ____

Tâche 2 : ____________________ **Port de gants** ☐oui ☐ non **Port de masque** ☐oui ☐ non

Produits utilisés : ____

Tâche 3 : ____________________ **Port de gants** ☐oui ☐ non **Port de masque** ☐oui ☐ non

Produits utilisés : ____

Tâche 4 : ____________________ **Port de gants** ☐oui ☐ non **Port de masque** ☐oui ☐ non

Produits utilisés : ____

Tâche 5 : ____________________ **Port de gants** ☐oui ☐ non **Port de masque** ☐oui ☐ non

Produits utilisés : ____

Tâche 6 : ____________________ **Port de gants** ☐oui ☐ non **Port de masque** ☐oui ☐ non

Produits utilisés : ____

Remarques :

Client 9 :

Tâche 1 : ____________ **Port de gants** ☐oui ☐ non **Port de masque** ☐oui ☐ non

Produits utilisés : ____________

Tâche 2 : ____________ **Port de gants** ☐oui ☐ non **Port de masque** ☐oui ☐ non

Produits utilisés : ____________

Tâche 3 : ____________ **Port de gants** ☐oui ☐ non **Port de masque** ☐oui ☐ non

Produits utilisés : ____________

Tâche 4 : ____________ **Port de gants** ☐oui ☐ non **Port de masque** ☐oui ☐ non

Produits utilisés : ____________

Tâche 5 : ____________ **Port de gants** ☐oui ☐ non **Port de masque** ☐oui ☐ non

Produits utilisés : ____________

Tâche 6 : ____________ **Port de gants** ☐oui ☐ non **Port de masque** ☐oui ☐ non

Produits utilisés : ____________

Remarques :

Client 10 :

Tâche 1 : ____________ **Port de gants** ☐oui ☐ non **Port de masque** ☐oui ☐ non

Produits utilisés : ____________

Tâche 2 : ____________ **Port de gants** ☐oui ☐ non **Port de masque** ☐oui ☐ non

Produits utilisés : ____________

Tâche 3 : ____________ **Port de gants** ☐oui ☐ non **Port de masque** ☐oui ☐ non

Produits utilisés : ____________

Tâche 4 : ____________ **Port de gants** ☐oui ☐ non **Port de masque** ☐oui ☐ non

Produits utilisés : ____________

Tâche 5 : ____________ **Port de gants** ☐oui ☐ non **Port de masque** ☐oui ☐ non

Produits utilisés : ____________

Tâche 6 : ____________ **Port de gants** ☐oui ☐ non **Port de masque** ☐oui ☐ non

Produits utilisés : ____________

Remarques :

Autres remarques :

Les données à caractère personnel communiquées dans le cadre de la recherche sont destinées uniquement au personnel habilité de l'INRS qui est responsable du traitement. Les données sont utilisées uniquement pour permettre de réaliser une étude épidémiologique concernant l'exposition professionnelle des coiffeuses au résorcinol et aux perturbateurs endocriniens et les effets sur la fonction thyroïdienne. L'INRS s'engage à ne pas transmettre ni vendre ces données à un tiers.
En application de la législation en vigueur vous disposez d'un droit d'accès, de rectification et d'effacement de vos données personnelles ainsi que des droits d'opposition au traitement de celles-ci. À ce titre, vous pouvez exercer ces droits en vous adressant au délégué à la protection des données de l'INRS : données.personnelles@inrs.fr. Vous avez également le droit d'introduire une réclamation auprès de la CNIL à l'adresse suivante : https://www.cnil.fr/fr/plaintes.
